# AAK1‐like: A putative pseudokinase with potential roles in cargo uptake in bloodstream form *Trypanosoma brucei* parasites

**DOI:** 10.1111/jeu.12994

**Published:** 2023-08-07

**Authors:** Jennifer A. Black, Christen M. Klinger, Leandro Lemgruber, Joel B. Dacks, Jeremy C. Mottram, Richard McCulloch

**Affiliations:** ^1^ The Wellcome Centre for Integrative Parasitology, School of Infection & Immunity University of Glasgow Glasgow UK; ^2^ Department of Cell and Molecular Biology, Ribeirão Preto Medical School University of São Paulo São Paulo Brazil; ^3^ Division of Infectious Diseases, Department of Medicine, Li Ka Shing Centre for Health, Research Innovation University of Alberta Edmonton Alberta Canada; ^4^ Glasgow Imaging Facility, School of Infection & Immunity University of Glasgow Glasgow UK; ^5^ Institute of Parasitology, Biology Centre Czech Academy of Sciences Ceske Budejovice (Budweis) Czech Republic; ^6^ York Biomedical Research Institute and Department of Biology University of York York UK

**Keywords:** AAK1, African trypanosomes, AP‐2 complex, endocytosis

## Abstract

Selection and internalization of cargo via clathrin‐mediated endocytosis requires adaptor protein complexes. One complex, AP‐2, acts during cargo selection at the plasma membrane. African trypanosomes lack all components of the AP‐2 complex, except for a recently identified orthologue of the AP‐2‐associated protein kinase 1, AAK1. In characterized eukaryotes, AAK1 phosphorylates the μ2 subunit of the AP‐2 complex to enhance cargo recognition and uptake into clathrin‐coated vesicles. Here, we show that kinetoplastids encode not one, but two AAK1 orthologues: one (AAK1L2) is absent from salivarian trypanosomes, while the other (AAK1L1) lacks important kinase‐specific residues in a range of trypanosomes. These AAK1L1 and AAK1L2 novelties reinforce suggestions of functional divergence in endocytic uptake within salivarian trypanosomes. Despite this, we show that AAK1L1 null mutant *Trypanosoma brucei*, while viable, display slowed proliferation, morphological abnormalities including swelling of the flagellar pocket, and altered cargo uptake. In summary, our data suggest an unconventional role for a putative pseudokinase during endocytosis and/or vesicular trafficking in *T. brucei*, independent of AP‐2.

## INTRODUCTION

Salivarian trypanosomes, including *Trypanosoma brucei* subspecies, cause human and animal African trypanosomiasis, which have profound medical and economic impacts in Africa (Büscher et al., 2017; Giordani et al., 2016). These parasites transmit via an infected tsetse fly (genus *Glossina*) bite, during which the trypanosomes invade the bloodstream and other tissues (Capewell et al., 2016; Trindade et al., 2016). In mammals, *T. brucei* persists extracellularly, with bloodstream form (BSF) cell survival depending upon the expression of variant surface gycoproteins (VSGs), that form a protective surface coat and undergoes stochastic switches in composition to evade host adaptive immunity (Silva Pereira et al., 2022; Sima et al., 2022). The high rate of BSF endocytosis, coupled with the motility of the trypanosome's flagellum, drags antibody‐VSG complexes, via hydrodynamic forces, into the flagellar pocket (FP; Engstler et al., 2007), the only site of endo‐ and exocytosis in *T. brucei* positioned within the posterior of the cell (Field & Carrington, 2009; Halliday et al., 2021; Link et al., 2021). Once endocytosed, antibody‐VSG complexes traffic to the endosomal system for degradation or dissociation (Engstler et al., 2007; Field & Carrington, 2009; Halliday et al., 2021; Pal et al., 2003).

In eukaryotes, the plasma membrane performs endo‐ and exocytosis, either via well‐characterized clathrin‐mediated endocytosis (CME; Kaksonen & Roux, 2018), or less understood clathrin‐independent endocytosis (CIE; Lamaze et al., 2001; Sandvig et al., 2018). Clathrin itself is highly conserved across the Eukarya, only lacking in organisms with highly reduced genomes, like the microsporidia (Barlow et al., 2014). Clathrin is present in *T. brucei* and when targeted by RNAi (Allen et al., 2003; Hung et al., 2004), the cells are killed. In the BSF cells, endocytosis defects arise, including FP enlargement (the so‐called ‘BigEye’ phenotype), and in dividing cells, the daughter cell FP also becomes swollen (the so‐called ‘LittleEye’ phenotype). In the PCF cells, a wider range of aberrant morphologies manifest, attributed to disrupted endosomal sorting and Golgi and lysosomal trafficking (Allen et al., 2003).

In eukaryotes, there are five adaptor protein (AP) complexes (Dacks & Robinson, 2017) that, alongside structural proteins, coordinate transport processes like endocytosis and secretory trafficking. The AP‐2 complex is required for recognition of incoming cargo and CME (Beacham et al., 2019; Mettlen et al., 2018). Targeting AP‐2 to the membrane is thought to occur first by an interaction between the α subunit and phosphoinositides from the membrane bilayer (Collins et al., 2002; Höning et al., 2005). Subsequently, the conformation of the complex changes (Beacham et al., 2019; Collins et al., 2002; Henne et al., 2010; Jackson et al., 2010; Kadlecova et al., 2016; Kelly et al., 2014; Kirchhausen et al., 2014; Kovtun et al., 2022) and it becomes phosphorylated by a protein kinase (discussed below) to permit the binding of specific peptide motifs (such as Yxxφ and [DE]xxxL[LI]; Doray et al., 2007; Owen & Evans, 1998; Schmid et al., 2006) on incoming cargo, including the transmembrane transferrin receptor protein (Conner & Schmid, 2003). The AP‐2 complex performs various functions to aid cargo internalization via clathrin‐coated vesicles (CCVs). For instance, the β2 subunit directly binds the clathrin heavy chain, leading to the assembly of CCVs and their internalization. Once the cargo is secured in the clathrin‐coated pit (CCP), the pit internalizes and is separated (by scission) from the plasma membrane, a reaction often mediated by dynamin (Beacham et al., 2019; Cocucci et al., 2014; Praefcke & McMahon, 2004). Next, the clathrin coat of the internalized vesicle is likely to be disassembled to permit progression of the CCV into the endocytic pathway. Phosphorylation of the μ2 subunit in a clathrin‐modulated manner in humans (Conner & Schmid, 2003; Jackson et al., 2003) is carried out by two kinases; adapter associated kinase 1 (AAK1; Conner & Schmid, 2003), and cyclin‐G associated kinase (GAK; Umeda et al., 2000), both belonging to the diverse Numb‐associated kinase (NAK) family. Phosphorylation is thought to enhance AP‐2 affinity for membrane bound sorting signals and cargo (Höning et al., 2005; Morillon et al., 2005; Olusanya et al., 2001; Ricotta et al., 2002); without AAK1‐mediated phosphorylation uptake of cargo such as transferrin (Olusanya et al., 2001) or Hepatitis C virus (Neveu et al., 2015) is blocked. However, some data suggests phosphorylation could also inactivate AP‐2 activities (Conner & Schmid, 2002; Partlow et al., 2019; Taylor & Kornev, 2011).

Loss of whole AP complexes, or adaptin subunits from specific complexes, has been reported in some eukaryotes (Lee et al., 2015; Woo et al., 2015), including salivarian trypanosomes, which belong to the Kinetoplastida grouping. In salivarian trypanosomes, all AP‐2 subunits appear absent (Klinger et al., 2016; Manna et al., 2013), which has been argued is a consequence of VSG trafficking. Though *T. brucei* performs CME, the absence of AP‐2, as well as the identification of novel clathrin interacting proteins (Manna et al., 2017), are indicative of a divergent process in the salivarian trypanosomes. However, a predicted kinase in *T. brucei* with homology to AAK1 was shown to interact with clathrin and localize at the posterior end of the cell, around the FP (Manna et al., 2017). Why an AAK1 orthologue might be retained in the absence of AP‐2 is unclear. Initial RNAi reports against the predicted kinase in BSF cells did not significantly alter cell proliferation, cell morphology, or cargo uptake, unlike RNAi against other clathrin interactors (Manna et al., 2017). However, subsequent reports of RNAi targeting the putative kinase (referred to as NAK/Tb927.9.6560) describe reduced BSF proliferation, and the appearance of aberrant cells (Jones et al., 2014; Stortz et al., 2017).

Here, we report phenotypic characterization of null mutants of the *T. brucei* AAK1 orthologue, which we named TbAAK1L1, to reflect new phylogenetic analysis of AAK1 distribution, in which we describe a previously unreported second AAK paralogue, AAK1L2, among kinetoplastids. AAK1L2 is absent from salivarian trypanosomes but is found in non‐salivarian trypanosomes and other kinetoplastids. We show that AAK1L1 displays sequence divergence in salivarian trypanosomes, including the absence of several kinase specific motifs, suggesting an absence of enzymatic activity. Nonetheless, TbAAK1L1 null mutants display reduced proliferation, the appearance of abnormal cells and altered cargo uptake, reminiscent of clathrin loss in *T. brucei* BSF cells.

## MATERIALS AND METHODS

### Homology searching

Predicted proteomes were downloaded for local BLAST searches; a complete list is provided in Table S1. All the BLASTp searches were performed using BLAST v2.2.29+ (Altschul et al., 1997), with an e‐value cut‐off of 0.05. All potential hits in the forward searches were subjected to reciprocal BLASTp searches against the original query proteome. Hits were considered true homologues if they hit the original query, or known homologues thereof, first, with an e‐value at least two orders of magnitude greater than the first non‐homologous hit.

### Alignment and phylogenetic analysis

Alignments were performed using the L‐INS‐I option of MAFFT v7.221 with default settings (Katoh & Standley, 2013) and trimmed manually. All the masked alignments are available on request. ProtTest v3.4.2 (Darriba et al., 2011) was used to find the optimal model of sequence evolution, starting with a maximum‐likelihood tree, and ignoring invariant sites and empirical base frequencies. Maximum‐likelihood reconstruction used RAxML v8.2.10 (Stamatakis, 2014). Inference of the larger alignment (Figure S1) used the –f a option for both tree topology and bootstrapping under the LG+Γ model, while inference of the kinetoplastid‐only alignment used the –f b option for bootstrapping only under the JTT+Γ model; in both cases, 100 bootstrap replicates were specified. Bayesian inference used MrBayes v3.2.6 (Ronquist et al., 2012), with 2 runs comprising 4 chains each with 1,000,000 MCMC generations and a sampling frequency of 500. A mixture of fixed amino acid models (prset aamodelpr = mixed) was used. The likelihood plot was inspected to ensure runs had reached a stationary plateau and the first 20% of sampling points were discarded as burnin (convergence statistics: average standard deviation of split frequencies of 0.015, average parameter value PSRF of 1.001). Mapping bootstrap support for bipartitions in the Bayesian topology was performed using the SumTrees program of the DendroPy package (both v4.1.0; Sukumaran & Holder, 2010). All the inference used gamma‐distributed rates with four categories and was carried out using the CIPRES web portal (Miller et al., 2010). Tree files were visualized using FigTree v1.4.2 (http://tree.bio.ed.ac.uk/software/figtree/) and manually edited using Adobe Illustrator.

### Nucleotide alignment and selection testing

A protein alignment was built as above and used to align nucleotide sequences using TranslatorX (Abascal et al., 2010). Statistical testing for relaxed selection used RELAX (Wertheim et al., 2015), testing for episodic diversifying selection used BUSTED (Murrell et al., 2015), while dN/dS calculations used SLAC (Kosakovsky Pond & Frost, 2005). All the testing was performed using the Datamonkey server (Delport et al., 2010) with default parameters.

### Motif logos

Motif logos were generated using WebLogo v2.8.2 (Crooks et al., 2004) with default settings.

### Cell culture and transfection

BSF Lister 427 parasites were cultured as described (Hirumi & Hirumi, 1989). Antibiotics were used at the following concentrations where appropriate: Blasticidin (BSD) 10 μg mL^−1^ and G418 2.5 μg mL^−1^. For transfection, ~3 × 10^7^ cells were transfected with 5 μg of linearized plasmid DNA using an AMAXA nucleofection system using human T‐cell nucleofection buffer (Lonza) as described (Devlin et al., 2016). Clonal populations were obtained by serial dilution and recovered 6 days after antibiotic addition. Genomic DNA was prepared from ~5 × 10^6^ cells using the Qiagen DNeasy Kit (Qiagen) as per manufacturer's instructions. All growth curves and cell cycle analyses were performed as described previously (Jones et al., 2014; Stortz et al., 2017). AAK1L1‐targeting RNAi cells originated from (Jones et al., 2014; Stortz et al., 2017).

### Endogenous epitope tagging

To endogenously epitope tag the C‐terminal locus of TbAAK1L1 with 12myc, a construct derived from the pNATx12myc plasmid was used (Alsford & Horn, 2008). A region of the ORF, from the 3' end excluding the stop codon, was amplified by PCR using the following primers: Forward, GTATAAGCTTCTACTGCGAGCAACCAAA and Reverse, GCTATCTAGACTTGAAGAGACTGGCGGA. The forward primer contains a HindIII restriction site, and the reverse contains an XbaI site. The plasmid was linearized prior to transfection and transfected BSF cells were selected for in BSD.

### Generation of *TbAAK1L1* null mutant cell lines

Heterozygote (+/−) and homozygous (*−/−*) deletion mutants of *TbAAK1L1* were generated by replacement of the majority of the ORF of *TbAAK1L1* with a drug resistance cassette. The plasmid pmtl23 (gift, Marshall Stark, University of Glasgow) was modified to contain a BSD or neomycin resistance gene. Cloning was performed as described (Devlin et al., 2016). The required fragments were amplified by PCR using the following primers: 5′ Region Forward, GCACGAAGCTT GCGGCCGCTGAAAAGGAGGGACAGGAA and Reverse, GCACGTCTAGAAGCACCTTCTCACTTAACC and 3′ Region Forward, GCACGGAGCTCACCAATGCAAACTCCACA and Reverse, GCACGATCGATGCGGCCGCGGATGTGATTGAGAATGGG. The plasmid was linearized with NotI prior to transfection. The Neomycin resistance plasmid was transfected first. The following selective drugs were used: G418 5 μg mL^−1^ or BSD 10 μg mL^−1^. Integration into the endogenous locus was confirmed using the following primers: 3′ UTR integration BSD Forward, (22) GGCCAAGCCTTTGTCTCAAG and Reverse, (137) CGGAGGGACGTAATAATA; 5′ UTR integration BSD Forward, (138) GGTGCAACTCTTTTGGTAA and Reverse, (166) GGGTGGATTCTTCTTGAGAC; 3′UTR integration NEO Forward, (126) GCTTGCCGAATATCATGG and Reverse, (137) same as for 3′ UTR reverse BSD; 5′ UTR integration NEO Forward, (167) GCGTGCAATCCATCTTGTTC and Reverse, (138) GCGTGCAATCCATCTTGTTC, ORF PCR Forward, (139) GGAAAACAAGGCATCTGC and Reverse, (140) TCCATTTCCCCATCCCAT. Numbers in brackets refer to the primer identification number.

### Immunofluorescence analysis

Approximately 2 × 10^6^ cells were harvested by centrifugation (405 *g* for 10 min). The pellet was washed in PBS by centrifugation (405 *g* for 3 min), the supernatant removed, and the pellet resuspended in ~50 μL PBS. The cells were settled for 5 min on a 12 well glass slide (Menzel‐Gläser) treated with Poly‐L‐Lysine (Sigma). The supernatant was removed and 25 μL 4% formaldehyde (FA) was added for 4 min. The FA was removed, and the cells washed 3× in 50 μL PBS for 5 min. For detection of anti‐myc, the cells were permeabilised with 25 μL PBS/0.2% Triton X‐100 (ThermFisher) for 10 min then 100 mM glycine was added for 20 min. The wells were then washed 3× in PBS for 5 min. The wells were blocked for 1 hour (h) with 25 μL blocking solution (1% BSA [Sigma], 0.2% Tween‐20 in PBS) in a wet chamber. After, 25 μL of anti‐myc:FITC conjugate antiserum (1:500; Sigma) diluted in blocking solution was then added and incubated for 1 h in a wet chamber. The wells were then washed 2× with PBS for 5 min. To each well, 5 μL of DAPI (SouthernBiotech) was added and incubated at room temperature for 4 min. For staining requiring KMX‐1 antiserum, cells were harvested and fixed as above. The cells were then washed three times in PBS for 5 min after which they were stained as described above with the following modification: cells were blocked and the antibody (α KMX1; 1:100; Hammarton Lab, University of Glasgow) diluted in 1% BSA only.

### Immunoblotting

Approximately 2.5 × 10^6^ cells were harvested by centrifugation, the supernatant removed, and the pellet re‐suspended in 10 μL 1× protein loading buffer (PLB: 250 μL 4× NuPAGE® LDS sample buffer [Invitrogen], 750 μL 1× PBS and 25 μL β‐mercaptoethanol) and denatured at 100°C for 10 min. Whole cell lysates were separated by SDS–PAGE using 10% Bis‐Tris NuPAGE® Novex® pre‐cast gels as per the manufacturer's instructions. Proteins were blot onto PVDF membrane. The proteins from the SDS–PAGE gel were transferred using a Mini Trans‐Blot® Cell (Bio‐Rad). The PVDF (Amersham Bio) membrane was immersed completely in 100% methanol for 1 min prior to submersion in transfer buffer (25 mM Tris pH 8.3, 192 mM Glycine and 20% [v/v] methanol). The filter paper, foam and the gel(s) were also equilibrated in transfer buffer for 10 min and the transfer performed by electrophoresis at 100 V for 120 min. The membrane after transfer was incubated for 10 min in the dark with Ponceau‐S solution (Sigma) to confirm protein transfer. After, membranes were washed once in PBST (PBS, 0.01% Tween‐20 [Sigma]) for 10 min on a rocker then the membrane was incubated for 1 h in blocking solution (PBST, 5% milk powder [Marvel]) or if required, overnight at 4°C. After, the membrane was rinsed once for 10 min in PBST then placed in blocking buffer containing the required primary antisera (1:7000 anti‐myc and 1:20,000 EF1α; Millipore) for 1 h. The membrane was then rinsed once in PBST for 20 min then placed in blocking solution containing the appropriate secondary antisera (1:3000 α mouse HRP conjugate and 1:5000 α rabbit HRP conjugate; ThermoFisher) for 1 h. After, the membrane was washed in PBST for 30 min then the SuperSignal West Pico Chemiluminescent Substrate (ThermoFisher) was pipette evenly onto the membrane and incubated in the dark for 5 min. The membrane was then exposed to an X‐ray film (Kodak) and the film developed using a Kodak M‐25‐M X‐omat processor.

### Transmission electron microscopy

Approximately 5 × 10^6^ cells were fixed in 2.5% glutaraldehyde and 4% PFA in 0.1 M sodium cacodylate buffer (pH 7.2) then post‐fixed for 1 h in 1% osmium tetroxide in 0.1 M sodium cacodylate buffer in the dark. The cells were washed several times with 0.1 M cacodylate buffer, and the samples stained (en bloc) with 0.5% aqueous uranyl acetate for 30 min, followed by dehydration in ascending acetone solutions (30%, 50%, 70%, 90%, and 100%). The samples were then embedded in Epon resin and sectioned (ultrathin sectioning, 60 nm thick). The samples were visualized on a Tecnai T20 transmission electron microscope (FEI) operating at 120 kV.

### Cryo‐immunolabeling

Approximately 5 × 10^6^ cells were fixed in 4% PFA and 0.2% glutaraldehyde in 0.1 M phosphate buffer (pH 7.2) then all the samples were infiltrated in 2.1 M sucrose overnight. Afterward, samples were subject to rapid freezing by immersion in liquid nitrogen. Cryosections were prepared at −100°C using an ultracut cryo‐ultramicrotome (Leica). Next, cryosections were blocked in 3% BSA in phosphate buffer then incubated with the primary antibody (anti‐myc; Millipore: 1: 7000) diluted in blocking buffer for 1 h. Cryosections were then washed several times in blocking buffer and incubated for 1 h with 10 nM gold‐labeled anti‐mouse antiserum (1: 20; Aurion) diluted in blocking buffer. Images were visualized as described above.

### Tomato lectin and Concanavalin a uptake by microscopy

For tomato lectin (TL) assays, ~1.5 × 10^6^ cells were collected by centrifugation at 500 *g* for 5 min (4°C). The pellet was re‐suspended in 1 mL ice cold 1× PBS, then washed by centrifugation as above and re‐suspended in 100 μL 1× PBS. A poly‐L‐lysine slide was prepared on which the cells were settled for 10 min at 4°C. The supernatant was removed and 25 μL of TL:FITC conjugate (Sigma; in 1× PBS) was added. TL:FITC was prepared by diluting 17 μL of the stock solution in 200 μL 1× PBS for usage. The slides were incubated in the dark at 4°C for 45 min. After this, the supernatant was removed, and the wells washed 2× with 1× PBS (cold; performed at 4°C). The cells were fixed at 4°C for 30 min in 3% FA in 1× PBS. Next, the wells were neutralized with 100 mM glycine (Sigma) and then stained with DAPI. For Concanavalin A (ConA), ~2 × 10^6^ cells were collected by centrifugation at 3287 *g* for 1 min at room temperature. The pellet was resuspended in 1 mL serum free HMI‐9 (1% BSA) and the cells were then incubated at 37°C for 20 min. Ten microliters of ConA (5 mg mL^−1^ stock, Concanavalin A Alexa Fluor™ 594 conjugate; ThermoFisher) was added and the cells incubated at 37°C for 30 min. The cells were then washed once in PBS (ice cold) and then fixed in 3% formaldehyde in PBS for 1 h at 4°C. The cells were washed in PBS, the supernatant removed and 20 μL of cells placed on a poly‐L‐lysine‐treated slide and left to air dry for 30 min. The slide was then placed in ice cold methanol for 30 min, the cells rehydrated in PBS for 5 min, then 5 μL DAPI Fluoromount‐G (Southern Biotech) was added to the cells, a coverslip added, and the slide sealed with nail varnish.

### Fluorescence microscopy

For images captured on an Axioskop 2 (Zeiss) fluorescence microscope a 63×/1.25 Oil Plan‐Neoflua objective and ZEN blue software (Zeiss) was used. For images captured on an Olympus IX71 DeltaVision Core System (Applied Precision, GW), a 1.40/100 × Oil objective lens was used; here, images were acquired using the SoftWoRx suite 2.0 software (Applied Precision, GE). Z‐stacks of varying thickness were acquired (no more than 10 μm) and the images de‐convolved (conservative ratio; 1024 × 1024 resolution) by the SoftWoRx software. Super‐resolution images were captured on an Elyra PS.1 super resolution microscope (Zeiss) using the structured illumination microscopy technique (SR–SIM). For all the images, a 63×/1.4 Oil PlanApo objective was used. Z‐stacks were captured using ZEN Black software (Zeiss), with a total Z‐thickness of ~7 μm. The images were reconstructed using automated set‐up and then aligned to the channel alignment files generated on the day of imaging. Final images were generated by merging the Z‐stacks then subsequently processed in ImageJ/Fiji (http://fiji.sc/Fiji; Schindelin et al., 2012). For most images, both the contrast and brightness of the DAPI signal was enhanced to improve visualization. For all images, the background was subtracted, and suitable false colors were assigned to the fluorescence channels. 3D rendered models were generated using the IMARIS software (http://www.bitplane.com/imaris/imaris; V.8.2). Scale bars are as stated in the figure legend. Image brightness was enhanced for clear visualization when required.

### Tomato lectin and Concanavalin a uptake by flow cytometry

For TL assays performed on live cells by flow cytometry, ~2 × 10^6^ cells were collected by centrifugation (cold) at 151 *g* for 10 mins (4°C). The pellet was re‐ suspended in 1 mL ice cold 1 × PBS, then washed by centrifugation as above and re‐suspended in 100 μL 1 mL serum free HMI‐9 (0.5 g mL^−1^ BSA). Five micrograms per milliliter of TL:FITC were added (Sigma; in 1× PBS) and the cells incubates at 4°C in the dark for 30 min. After, the cells were washed with 1× HBS (1% FBS, 50 mM HEPES, 50 mM NaCl, 5 mM KCl, 70 mM Glucose, pH 7.5) then re‐suspended in 1× PBS containing propidium iodide (PI; 10 μg mL^−1^) for 10 min in the dark. All the samples were filtered through a 35 mm nylon mesh membrane and ran on a FACSCelesta (BD Biosciences). Flow cytometry data was analyzed in FlowJo v10 (https://www.flowjo.com). An unstained and secondary controls were prepared for each experiment. To generate a ‘dead’ control, prior to PI staining, cells were heated to 70°C for 3 min, which in our hands, routinely killed up to 90% of the cell population. Viability across the cell lines was routinely over 90%. Approximately 20,000–25,000 events were analyzed per sample. For endocytosis assays using ConA or TL:FITC on live cells by flow cytometry at 37°C, ~2 × 10^6^ cells were collected by centrifugation at 3287 *g* for 1 min at room temperature. The pellet was resuspended in 1 mL serum free HMI‐9 (1% BSA) and the cells were then incubated at 37°C for 10 min. Two microliters of ConA (5 mg mL^−1^ stock, Concanavalin A Alexa Fluor™ 594 conjugate; ThermoFisher) was added and the cells incubated at 37°C for a further 30 min. Immediately after the 30 min incubation, cells were placed on ice for 3 min then washed in PBS (ice cold), then resuspended in 1 mL PBS containing 5 μg DAPI. Cells were filtered and analyzed as described for TL. An unstained and 594 secondary controls were prepared for each experiment. To generate a ‘dead’ control, prior to DAPI staining, cells were heated to 70°C for 3 min, which in our hands, routinely killed ~70%–90% of the cell population. Viability across the cell lines was routinely between 80% and 99%. Approximately 30,000–40,000 events were analyzed per sample.

### Cytoskeletal extractions

Approximately 2 × 10^6^ cells were harvested by centrifugation (1000 *g* for 10 min) then washed in PBS. The supernatant was removed, and the cells re‐suspended in 25 μL 0.25% NP40 in 100 mM PIPES (Sigma) with 1 mM MCl_2_ (Sigma) pH 6.8 then settled onto a poly‐L‐lysine treated slide for 10 min. The supernatant was removed, and the cells washed 2 × 5 min in 100 mM PIPES with 1 mM MCl_2_ (pH 6.8). After the final wash, the supernatant was removed, and the cells fixed in 3% FA in PBS for 10 min followed by neutralization using 100 mM glycine for 5 min (2×). After, the glycine was removed, and the cells washed in 1× PBS. The slides were then placed in a wet chamber and incubated with anti‐myc antiserum as detailed in the immunofluorescence analysis section, followed by DAPI staining.

### Graphical presentation and statistical analysis

All the graphical information and appropriate statistical testing were performed using GraphPad Prism v9.0 and Microsoft Excel. Statistical tests are as described in the figure legends and are denoted by an asterisk (*). Figures were created with BioRender.com. Dissociation predictions were performed using IUPred2A (Mészáros et al., 2018; https://iupred2a.elte.hu) and the model shown in Figure S2D using Phyre2 (Kelley et al., 2015).

## RESULTS

### TbAAK1L1 is the sole homologue of numb‐associated protein kinases (NAKs) in *T. brucei*

**FIGURE 1:**
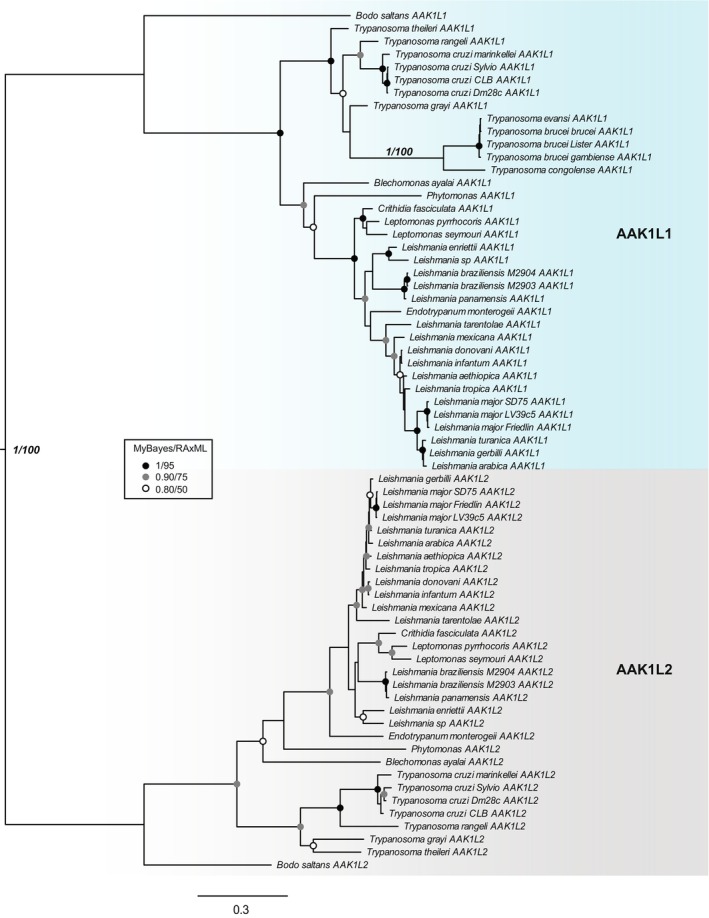
Phylogenetic analysis of two AAK1 paralogues in kinetoplastids: AAK1L1 and AAK1L2. Separation of kinetoplastid NAK family members into two distinct clades, AAK1L1 and AAK1L2, is shown, with strong phylogenetic support. Inference is based on a 67‐taxon by 329‐position alignment, and the best Bayesian topology is shown. Posterior probabilities and bootstrap support values are provided for two important nodes; support for all other nodes is denoted by symbols as per figure inset, whereby the value in both methods is at least that shown for the relevant symbol.

We began our characterization of TbAAK1L1 by confirming its putative identity as an ortholog of AAK1. A BLASTp search of TbAAK1L1 (Tb927.9.6560) against the non‐redundant database (NRDB) limited to *Homo sapiens* (taxid 9606) revealed HsAAK1 (NP_055726.3) as the first hit, with an e‐value of 1e‐24. However, a reciprocal BLASTp search using this query against the *T. brucei* proteome at tritrypdb.org hit a putative Nek/NIMA‐related protein kinase (Tb927.10.460, hereafter referred to as TbNekL) before TbAAK1L1 (e‐value difference 5e‐27 vs. 9e‐25). Since this is less than two orders of magnitude difference between the best and send‐best scoring candidate orthologues, the difference is inconclusive and so we used phylogenetics to resolve the relationships. We carried out additional reciprocal BLASTp searches using TbAAK1L1, TbNekL, and HsAAK1 against a dataset of 73 genomes and transcriptomes representing a broad swathe of eukaryotic diversity (Table S1). A maximum‐likelihood tree of all putative homologues revealed a clear split between NIMA/Nek‐like and AAK1‐like (NAK family) kinases (bootstrap support, BS = 100), with TbAAK1L1 grouping with other NAKs and TbNekL grouping with NIMA/Nek‐like members, as expected (Figure S1). The identity of TbAAK1L1 as an AAK1 homologue is further supported by its domain structure compared with the human homologue (Figure S1). We noticed a clear grouping of kinetoplastid NAK homologues into two distinct paralogues (BS = 63, Figure S1), which we refer to as AAK1L1 and AAK1L2. Comparison of these revealed that AAK1L1 orthologues are approximately twice the length of AAK1L2 (roughly 700 vs. 350 amino acids), with the main difference being the length of the variable C‐terminal region. Although some paralogues were initially incomplete or missing, we were able to complete these gene models with tBLASTn searches using the most closely related paralogue as a complete gene model (Table S1). However, we were unable to identify any orthologues of AAK1L2 in salivarian trypanosomes. Restricting the dataset to kinetoplastid homologues only and using both Bayesian and maximum‐likelihood reconstruction (materials and methods) strongly supports the split between paralogues (posterior probability, PP = 1, BS = 100, Figure 1), suggesting that AAK1L2 was secondarily lost in salivarian trypanosomes. Furthermore, the long internal branch separating salivarian from non‐salivarian trypanosome AAK1L1 orthologues (Figure 1) suggests that the former represent comparatively divergent sequences in the salivarian AAK1L1 proteins.

**FIGURE 2:**
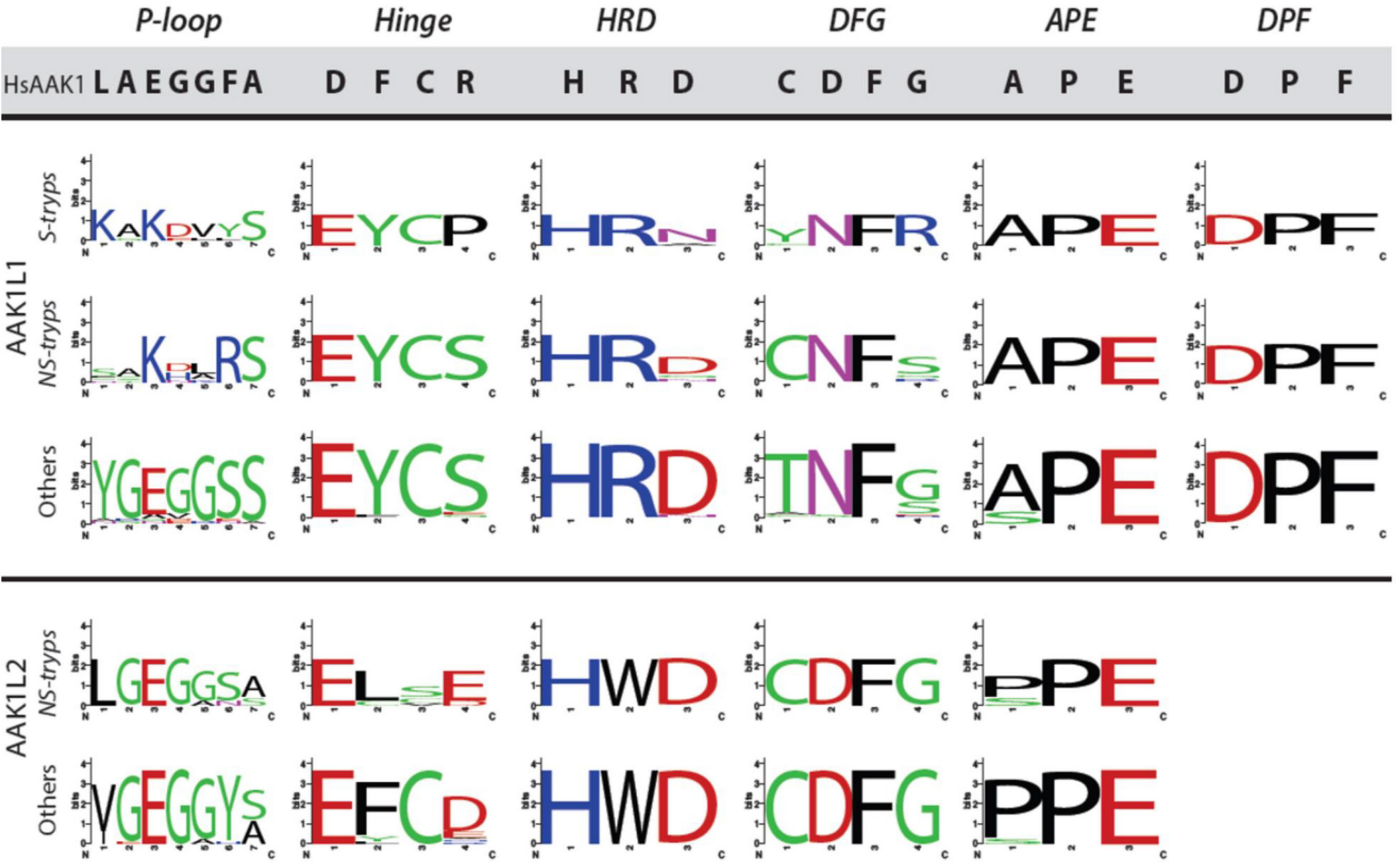
Conserved motifs in AAK1L1 and AAK1L2 homologues. This figure shows sequence logo representations of conserved motifs across groups of taxa compared with the corresponding sequence in *Homo sapiens* AAK1. The p‐loop (phosphate binding), hinge (structural), and HRD, DFG, and APE (kinase activity and regulation) motifs are present within the N‐terminal catalytic kinase domain 31, while the DPF motif (interaction with AP‐2 α) is present near the C‐terminus in each sequence47. Each column represents one motif, labeled at the top in bold italics. The corresponding sequence in HsAAK1 is provided in bold font against a gray background. For each group (salivarian trypanosomes, ‘S‐tryps’; other trypanosomes, ‘NS‐tryps’; and *Leishmania* spp. and other related taxa, ‘Others’), the corresponding sequence logo is shown. Solid black line separates AAK1L1 from AAK1L2 homologues.

To further investigate the putative divergence of salivarian trypanosome AAK1L1 paralogues, we examined key conserved amino acid residues within the predicated kinase domain (Kanev et al., 2019) of AAK1 paralogues across three kinetoplastid groups (salivarian trypanosomes, all other trypanosomes, *Leishmania* and related taxa except *B. saltans*) and compared them to the homologous residues in HsAAK1 (Sorrell et al., 2016; Figure 2). We tested for conservation of the p‐loop (required for phosphate binding), the hinge region (which connects both lobes of the kinase domain), and for the presence of HRD, DFG, and APE motifs. The HRD and DFG motifs are required for catalysis in catalytically competent kinases and the APE motif stabilizes the kinase activation loop (AL), which is involved in substrate binding (Kanev et al., 2019). Although some motifs were well conserved in AAK1L1 from salivarian and non‐salivarian trypanosomatids in comparison to HsAAK1 (e.g., the APE motif in AAK1L1 orthologues), we observed numerous differences between the human and kinetoplastid sequences, including many non‐synonymous substitutions. For example, in all AAK1L1 orthologues, we detected the consistent replacement of the DFG motif aspartic acid (D) with asparagine (N) resulting in a predicted DFN motif. In addition, in salivarian trypanosomatids, we found a replacement of the HRD motif aspartic acid (D) with an asparagine (N) generating an HRN motif. Moreover, all the AAK1L1 orthologues except those in the non‐salivarian trypanosomes encode a non‐synonymous amino acid in place of the active‐site cysteine (either tyrosine or threonine, Figure 2). Such conserved catalytic residues are commonly mutated in protein pseudokinases, which are pseudoenzymes that lack canonical phosphotransferase activity but possess a potential wealth of other functions (Kwon et al., 2019; Mace & Murphy, 2021). Thus, AAK1L1 is likely a pseudokinase in salivarian trypanosomatids. In all the AAK1L2 orthologues (absent from salivarian trypanosomatids), we found a replacement of the HRD motif arginine (R) with tryptophan (W) and a loss of the alanine (A) residue for a proline (P) in the APE motif (Figure 2 and Figure S2A), suggesting AAK1L2 kinase activity may also be altered. Surprisingly, a C‐terminal DPF motif, hypothesized to mediate interaction between AAK1 and the alpha subunit of AP‐2 (Conner & Schmid, 2002; Owen et al., 1999), is conserved across AAK1L1 orthologues but absent from AAK1L2 orthologues (Figure 2).

As salivarian trypanosomes lack AP‐2 (Klinger et al., 2016; Manna et al., 2013), their remaining AAK1 homologue may have undergone neofunctionalization and/or relaxed selection. Any of these scenarios would be consistent with the long internal branch (Figure 1) and non‐synonymous replacement of residues important for kinase function (Figure 2). To investigate this, we built a back‐translated nucleotide alignment of AAK1L1 orthologues and trimmed it such that only codons present in at least one representative of both groups (salivarian trypanosomes and all other kinetoplastids) were included, resulting in a final alignment of 36 taxa and 712 positions, and tested for relaxed selective pressure. Although the test parameter suggested relaxed selection (k = 0.85, i.e., k < 1), this was not significant (*p*‐value = 0.073). Comparatively, we found evidence for episodic diversifying selection in salivarian trypanosomes relative to all other AAK1L1 sequences (LRT, *p*‐value = 0.036). This finding suggests these sequences may have undergone diversification concurrent with the loss of AP‐2 and are retained in extant organisms due to their new functional role. To test whether these sequences are retained under negative selection, we calculated phylogenetic‐aware dN/dS values for all positions across either the whole alignment or considering both groups separately using SLAC (Table S2). Site‐wise dN/dS values across the entire phylogeny were typically below −0.5, indicating negative selection, except for a few clusters of positive values, most of which were in the C‐terminal half of the alignment (Figure S2B). A very similar pattern was observed considering either group separately (Figure S2B), suggesting that negative selection predominates for all the AAK1L1 orthologues.

These data show that TbAAK1L1 is the sole homologue of NAKs in *T. brucei* and other salivarian trypanosomes. Furthermore, despite the loss of AP‐2, a key interacting partner of AAK1 homologues, and frequent non‐synonymous replacement of functionally important residues (Figure S2B), AAK1L1 orthologues in salivarian trypanosomes appear to be under negative selection, albeit at a less intense level than other AAK1L1 orthologues. In support of TbAAK1L1 sharing homology to AAK1, further sequence analysis also revealed a highly disordered C‐terminal region, like HsAAK1L1 (Figure S2C,D).

### TbAAK1L1 localizes near to the FP of BSF *T. brucei*

**FIGURE 3:**
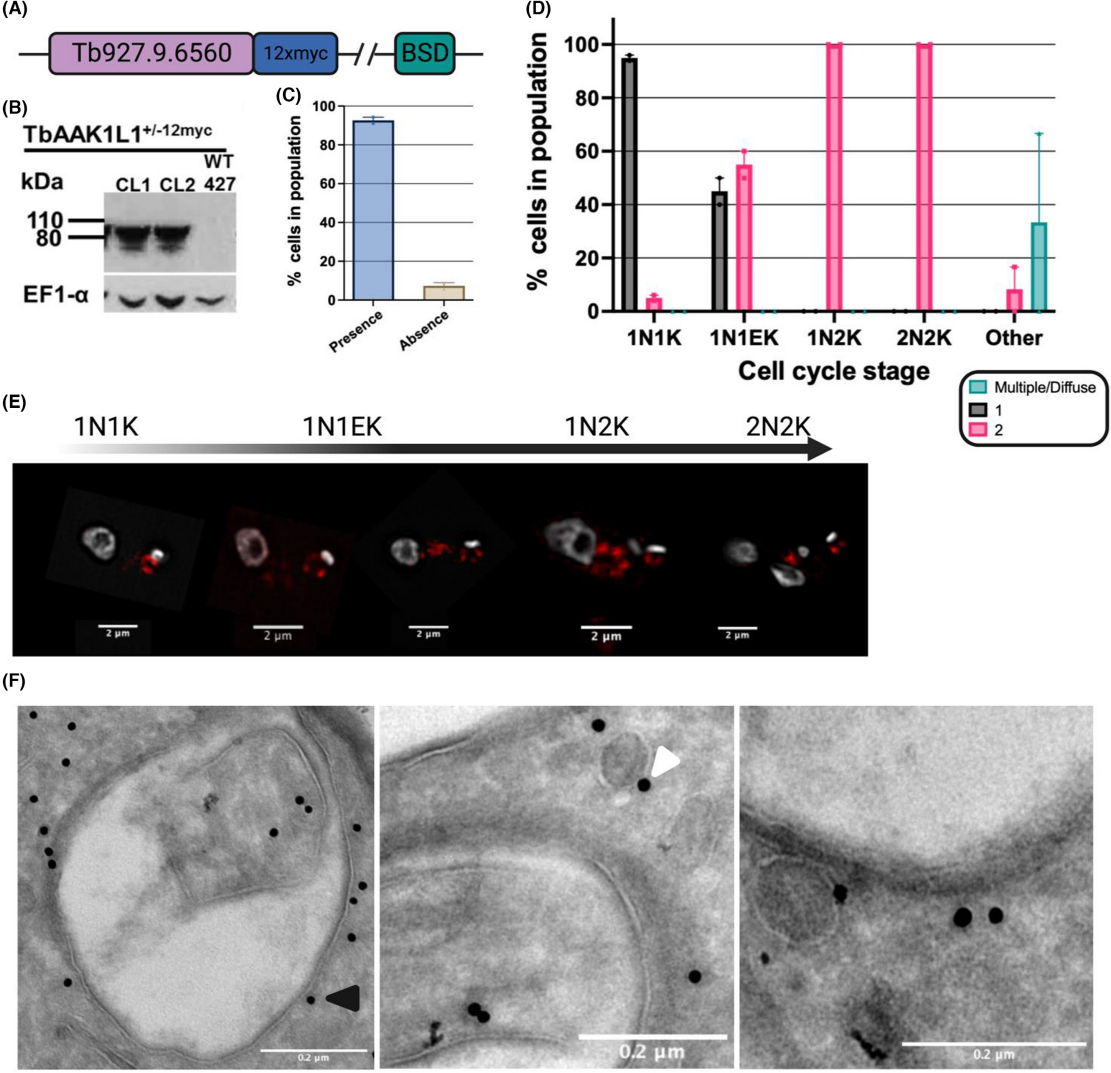
Epitope tagging of TbAAK1L1 reveals localization at the cell posterior. (A) Diagram illustrating the location of the 12xmyc tag inserted into the endogenous *TbAAK1L1* (Tb927.9.6560) locus; BSD (blasticidin). (B) Immunoblot showing endogenously 12myc tagged TbAAKL1 heterozygote cells (TbAAK1L1^+/−12myc^). Whole cell lysates were separated and blotted with antiserum recognizing myc and EF1α (as a loading control). Wild type (WT 427) cells were used as a negative, untagged control; sizes are shown in kDa. Predicted size (tritrypdb.org): 80.199 kDa + 14.4 kDa 12xmyc epitope; total estimated size: 94.599 kDa. (C) TbAAK1L1^+/−12myc^ CL1 cells were scored for the presence or absence of myc localisation signal. Fluorescent signal was set relative to background signal detected in WT untagged cells (control cell line). Over 100 cells were counted. Error bars (± SEM) are shown. *n* = 2 independent experiments. (D) Anti‐myc signal was scored in TbAAK1L1^+/−12myc^ CL1 cells for each cell cycle stage, as determined by DAPI staining (shown in gray). Anti‐myc signal was separated into three categories: one focus, two foci, or greater than two foci and/or a diffuse unspecific localization. Over 150 cells were counted/experiment. Error bars (± SEM); *n* = 2 independent experiments. (E) Representative high‐resolution images of the subcellular localisation of TbAAK1L1^+/−12myc^ in BSF cells throughout the cell cycle. Indirect immunofluorescence was performed using anti‐myc antiserum conjugated to Alexa Fluor 488 (red). DAPI was used to visualize the n‐ and kDNA (gray). Scale bar = 2 μm. Images were captured on a DeltaVision microscope. (F) Representative images of TbAAK1L1^+/−12myc^ localisation by ImmunoGold® labeling. The 12xmyc fusion protein was detected using 10 nm ImmunoGold® particles. Black arrow indicates a gold particle in contact with the FP membrane. A white arrow indicates a gold particle associated with a vesicle. Images were captured on a Tecnai T20 EM microscope. Images were processed in ImageJ and the scale bar added to all images (0.2 μm).

Endogenously tagged TbAAK1L1 localizes diffusely to the posterior end of BSF cells (Manna et al., 2017). However, in PCF cells, a single focus at the posterior end of the cell was observed only under starvation conditions (Fritz et al., 2015), postulated to localize to RNA stress granules. We independently assessed the subcellular localisation of TbAAK1L1 in BSF cells by endogenously tagging TbAAK1L1 with 12xmyc epitopes on the C‐terminus (Figure 3A). Modification of the endogenous locus was confirmed by PCR (Figure S3A) and expression of the fusion protein by immunoblotting (Figure 3B). The second TbAAK1L1 allele was disrupted with a drug resistance gene and confirmed by PCR (Figure S3A). Endogenously tagging TbAAK1L1 had no significant effect on parasite proliferation relative to wild type (WT) cells (Figure S3A). Immunofluorescence assays on TbAAK1L1^+/−12 myc^ parasites revealed that ~95% of BSF cells harbored discrete myc signal at the cell posterior and proximal to the kDNA (Figure 3C), as previously observed (Manna et al., 2017). In a subset of cells, we detected an additional focus of myc signal in the posterior of the cell. To ask if these different localisation patterns correlated with the cell cycle stage of the parasites, we scored cells based on their nuclear ‘N’ and kinetoplast ‘K’ configurations for the presence of 1, 2 or multiple/diffuse myc foci/signal (Figure 3D,E and Figure S3B). Diffuse refers to cells in which no discrete foci were seen. In cells with an n:k ratio of 1:1 (1N1K; G1 phase cells), ~95% had a single focus. In S‐phase cells (1N1EK cells), ~ 50% contained 2 discrete foci. These foci appeared as spatially distinct arrangements of the protein at either end of the dividing kDNA. In all cases, the anterior end of the kinetoplast appeared to harbor a larger concentration of the protein (Figure 3E and Figure S3B). Following kDNA segregation (1N2K cells; G2/M‐phase cells), 2 foci were observed in 100% of cells, largely reflecting the protein localisation in 1N1EK cells. After mitosis (2N2K), and prior to cytokinesis, these two foci of myc signal become approximately even sized across 100% of cells. This distinct pattern of localisation was lost in detergent‐extracted parasites (Figure S3C) suggesting TbAAK1L1 is unlikely to associate with the parasite cytoskeleton.

We next performed immuno‐EM on TbAAK1L1^+/−12myc^ cells using 10 nm ImmunoGold® particles (Figure 3F and Figure S4). Gold particles were detected within the cytoplasm at the posterior end of the parasite cell. In some cases, we detected gold particles in contact with, or proximal to, the cytoplasmic face of the FP membrane (Figure 3F; black arrow), and in others, gold particles were localized to small membrane‐bound structures, reminiscent of cytoplasmic vesicles and/or endosomes (Figure 3F; white arrow). Occasionally, gold particles co‐localized with flagellum itself and the kDNA.

Taken together, our data shows that TbAAK1L1 localizes in proximity to the cytoplasmic side of the FP membrane, and putatively to cytoplasmic vesicles in an organization that alters dynamically throughout the cell cycle of BSF *T. brucei*.

### TbAAK1L1 null mutants are viable in vitro, and its loss effects BSF *T. brucei* cell morphology

In one study (Manna et al., 2017), TbAAK1L1 depletion by RNAi did not alter BSF cell proliferation or morphology in vitro, whereas two other studies (Jones et al., 2014; Stortz et al., 2017) showed that depletion of TbAAK1L1 correlates with a loss of proliferation and the accumulation of aberrant cells. Due to these conflicting reports, we opted to determine the importance of the putative pseudokinase in BSF cells by generating null mutant cell lines.

**FIGURE 4:**
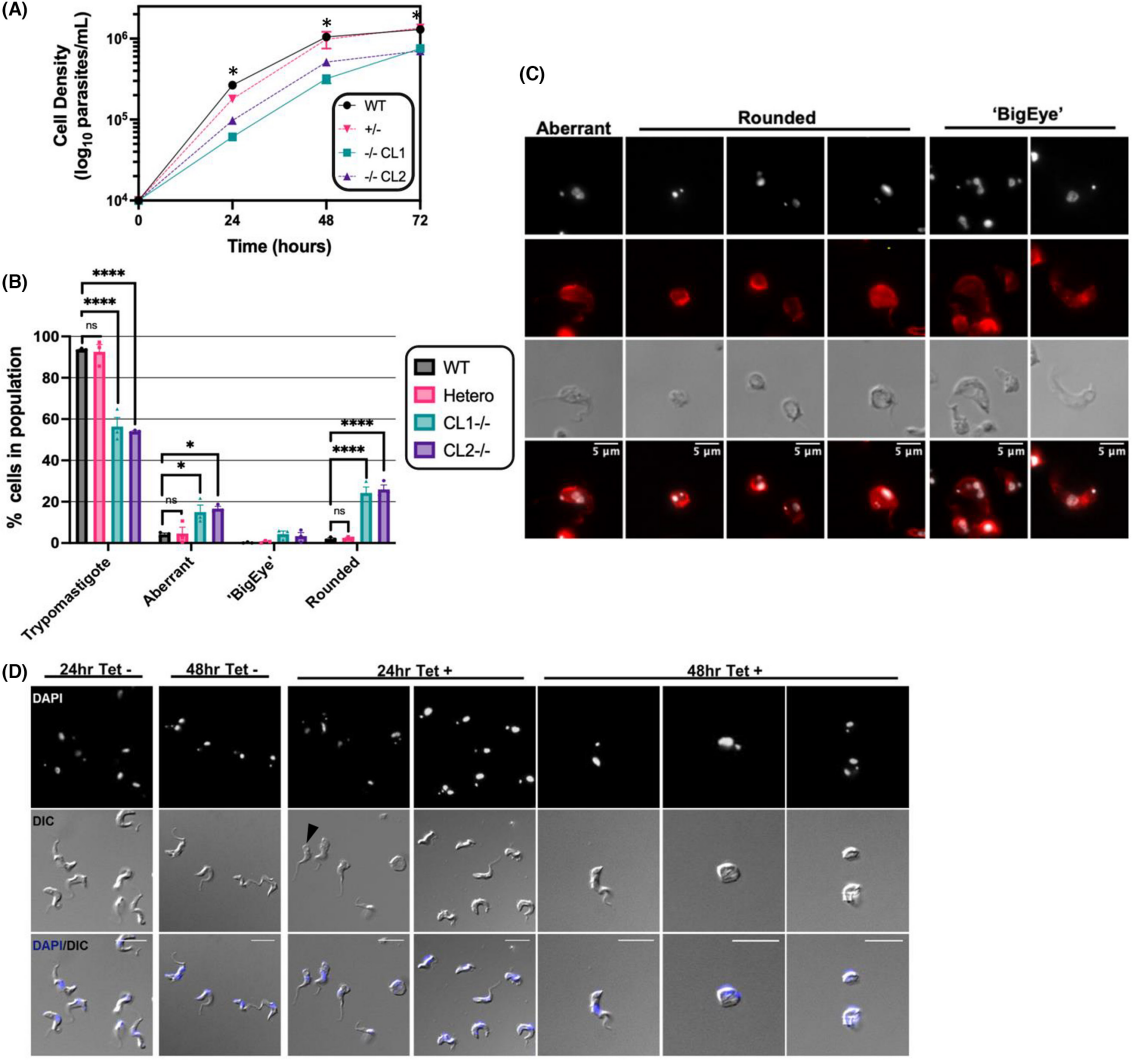
Deletion of TbAAK1L1 is associated with reduced BSF proliferation and the presence of morphologically aberrant cells in vitro. (A) Growth of two clones of *TbAAK1L1−/−* cells (CL1 and CL2) were compared with WT 427 and *TbAAK1L1*+/− cells. Error bars (± SEM); *n* = 3 independent experiments; (*) = *p* < 0.05. Statistical significance was calculated to compare WT cell density at each individual time point with the corresponding *TbAAK1L1−/−* clone (CL1 and CL2) densities using a Mann Whitney *U*‐test (one tailed). Each row was analyzed individually where a consistent standard deviation (SD) was not assumed. The growth of each clone was found to be significantly reduced relative to the WT control at all the three selected time points, thus only one single asterisk is shown. (B) Morphological analysis of WT, *TbAAK1L1−/−* (CL1 and CL2) and *TbAAK1L1+/*− cells in vitro. DAPI was used to visualize the n‐ and kDNA. The cells were categorized into the following categories based on their morphology: trypomastifgote, aberrant, ‘BigEye’, and rounded. The cells were counted and expressed as a percentage of the total population (over 200 cells/cell line/experiment). Error bars (± SEM); (*) = *p* < 0.005, (****) = *p* < 0.0001; *n* = 3 independent experiments. Statistical significance was calculated to compare WT 427 cell density at each individual time point with the corresponding *TbAAK1L1−/−* clone (CL1 and CL2) densities using a one‐way ANOVA using a Dunnett's multiple comparison post‐test. Each row was analyzed individually where a consistent standard deviation (SD) was not assumed. (C) Representative images of aberrant, rounded and ‘BigEye’ cells from *TbAAK1L1−/−* CL1. Cells were stained with DAPI (to visualize n‐ and kDNA; shown in gray) and α KMX‐1 (to visualize β tubulin; shown in red). Images were captured on an Axioskop2 (Zeiss). Scale bar = 5 μm. (D) Representative images of aberrant, cells following depletion of TbAAK1L1 by RNAi. RNAi cell line originates and is as described in Stortz et al. (2017) and Jones et al. (2014). Cells were stained with DAPI (to visualize n‐ and kDNA; shown in gray). Images were captured on an Axioskop2 (Zeiss). Scale bar = 10 μm. A Black arrow indicates a cell with the ‘BigEye’ phenotype.

We generated *TbAAK1L1* null mutants (*TbAAK1L1−/−*) by disrupting each allele with a drug resistance cassette (Figure S5A). Integration of each construct and disruption of the ORF was confirmed by PCR (Figure S5B) after clonal selection. Two independent clones (CL1, CL2) were chosen for further analysis. The successful ablation of both *TbAAK1L1* alleles (*TbAAK1L1−/−*) demonstrates the protein is non‐essential for BSF survival in vitro. Indeed, the null mutants were obtained and at a transfection efficiency that is typical for our lab. However, loss of TbAAK1L1 led to reduced proliferation relative to both WT and a heterozygote (+/−) mutant (Figure 4A). In addition, we observed a significant increase in cells with ‘aberrant’ nuclear:kinetoplastid (N:K) DNA configurations when examined by DAPI staining (~20% of the population; Figure S5C). Though such data could be suggestive of an altered cell cycle, the distorted morphology of the *TbAAK1L1−/−* cells could obscure visualization of the n‐ and kDNA, with such data most simply explained by wider changes in cell morphology.

To examine these morphological effects further, we scored cells based on their appearance (Figure 4B,C and Figure S5). Approximately 7% of WT cells and 3% of *TbAAK1L1+/*− cells appeared visually aberrant, in contrast to ~44‐46% of *TbAAK1L1−/−* cells. Visually aberrant cells were further categorized as aberrant (cells with no clear, definable defect), rounded (circular in shape) and ‘BigEye’ (cells with enlarged FPs but not rounded in shape; Allen et al., 2003). Representative images are shown in Figure 4C and Figure S5D. The number of trypomastigote‐shaped cells in both null mutants (~50% of cells) was significantly reduced relative to WT. The increase in morphologically aberrant *TbAAK1L1−/−* cells was primarily accounted for by a significant increase in ‘rounded’ cells (~20%–25% of all cells). In many cases, the flagellum was not clearly observed, or appeared short in length. The next most common category was aberrant, but not clearly rounded, cells (~10%–15% of cells). Finally, a small increase in cells with the ‘BigEye’ phenotype was observed in both mutant clones (~5% of cells) relative to WT. However, as demonstrated previously (Allen et al., 2003), cells with enlarged FPs are often fragile and fail to retain their structure following centrifugation. Thus, it is possible that many cells with these enlarged structures were lost during sample preparation. We wondered if these morphological changes were secondary effects due to the loss of TbAAK1L1 and, thus, reflect potential adaptions during in vitro culture. Using the RNAi cell line targeting TbAAK1L1 in BSF cells from our previous study (Stortz et al., 2017), revealed similar rounded and ‘BigEye’ cells at both 24 and 48 h post tetracycline induction (Figure 4D), suggesting these morphological changes arise early and are not a consequence of culture adaption.

**FIGURE 5:**
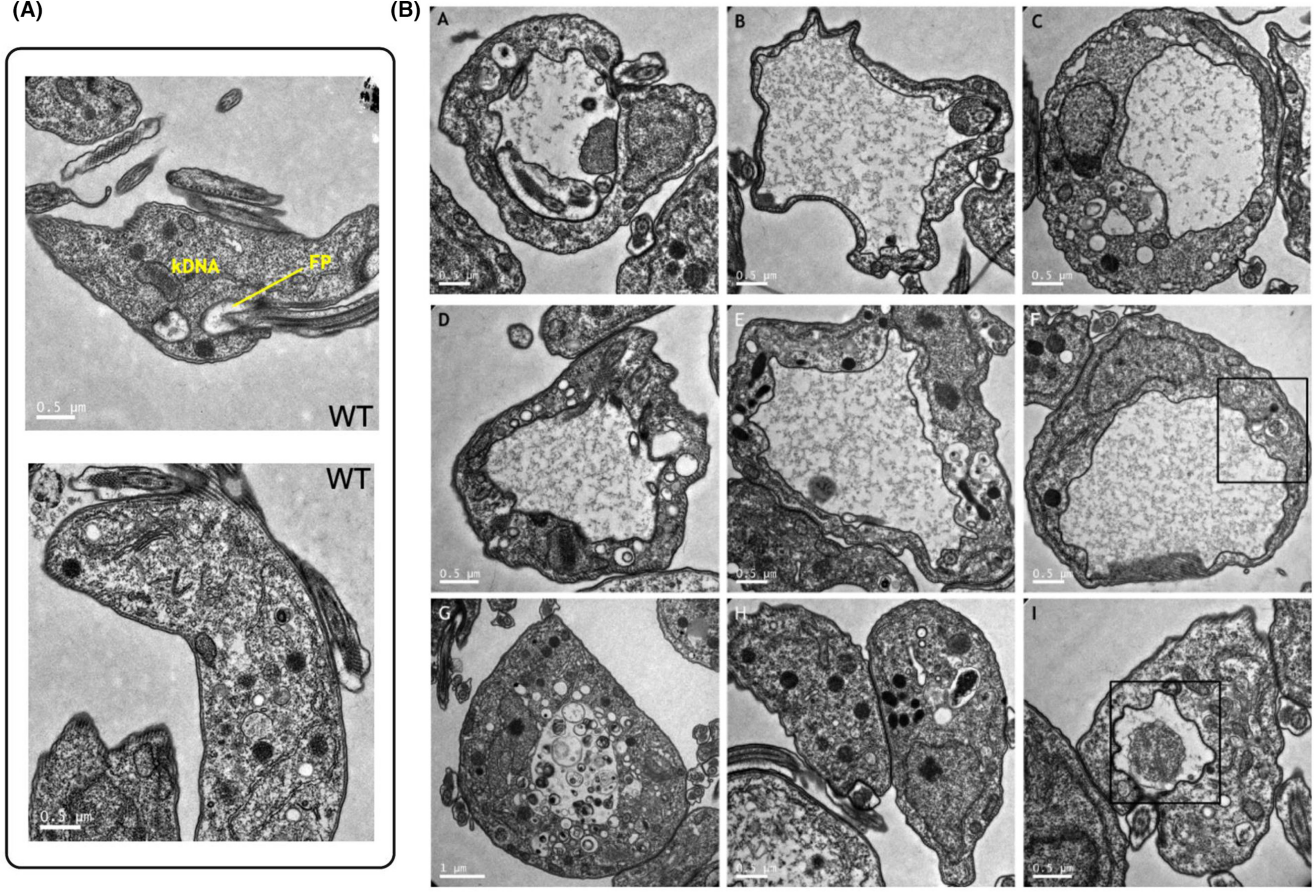
Deletion of TbAAK1L1 results in enlargement of the flagellar pocket. (A) Representative images of wild type (WT) cells fixed and imaged using a Tecnai T20 transmission electron microscope. Notable cell features within the posterior region of the cell are annotated: FP (flagellar pocket), kDNA (kinetoplast DNA). Scale bar = 0.5 μm. (B) *TbAAK1L1−/−* cells captured on a Tecnai T20 transmission electron microscope (TEM). Scale bars are as annotated on the images (range between 1 and 0.5 μm). Black boxes indicate regions enlarged in (Figure S5C).

To ask if the morphological changes were reflective of FP enlargement, we performed transmission electron microscopy (TEM) to visualize the ultrastructure of the FP of *TbAAK1L1−/−* cells (Figure 5 and Figure S5). Examples of WT and +/− cells are shown in Figure 5A and Figure S6A. Loss of TbAAK1L1 resulted in complex alterations, ranging in severity, in internal cellular morphology, though no clear kDNA or nDNA defects could be observed. In one cell, the nuclear membrane appeared disrupted; however, the phenotype of this cell was particularly severe and it is possible the general cell structure was breaking down (Figure 5B, panel G). Most cells (Figure 5B, panels A–I and Figure S6B) had an enlarged FP, as determined by the presence of several features: a glycan rich matrix within the perceived enlarged FP; identification of a flagellum entry point and an FAZ; and an electron dense coating on the inner surface of the membrane (characteristic of the VSG coat). In some cells, the FP encompassed almost the entire cell volume (Figure 5B, panels B and F), though in all cases the FP was not spherical in shape, instead appearing misshapen and folded. In some cases, densely coated objects were observed within the FP (Figure 5B, panel I; Figure S6C). Furthermore, swollen ‘vesicle‐like’ structures, some with internal cargo (occasionally very electron dense) and others devoid of content, could be discerned (Figure 5B, panels G–I). No clear defect in the flagellar pocket collar (FPC; the point at which the flagellum enters the FP) could be observed in any morphologically abnormal *TbAAK1L1−/−* cells (Figure S6D), though further and more quantitative approaches are required to confirm these observations. Though the preponderance of enlarged FPs in the TEM images appears distinct from the small number of cells seen with a ‘BigEye’ phenotype by light microscopy (Figure 4), it is possible that TEM preparation is more preserving of fragile cell structures. In addition, while true that a minority of cells had a ‘BigEye’ phenotype, ~20%–30% of the mutant population had a rounded appearance and, when assayed for tomato lectin uptake (see below), only some rounded cells also had enlarged FPs, meaning the ‘BigEye’ phenotype and enlarged FP may not always overlap in these mutants.

### TbAAK1L1 KO are defective in surface bound cargo uptake

**FIGURE 6:**
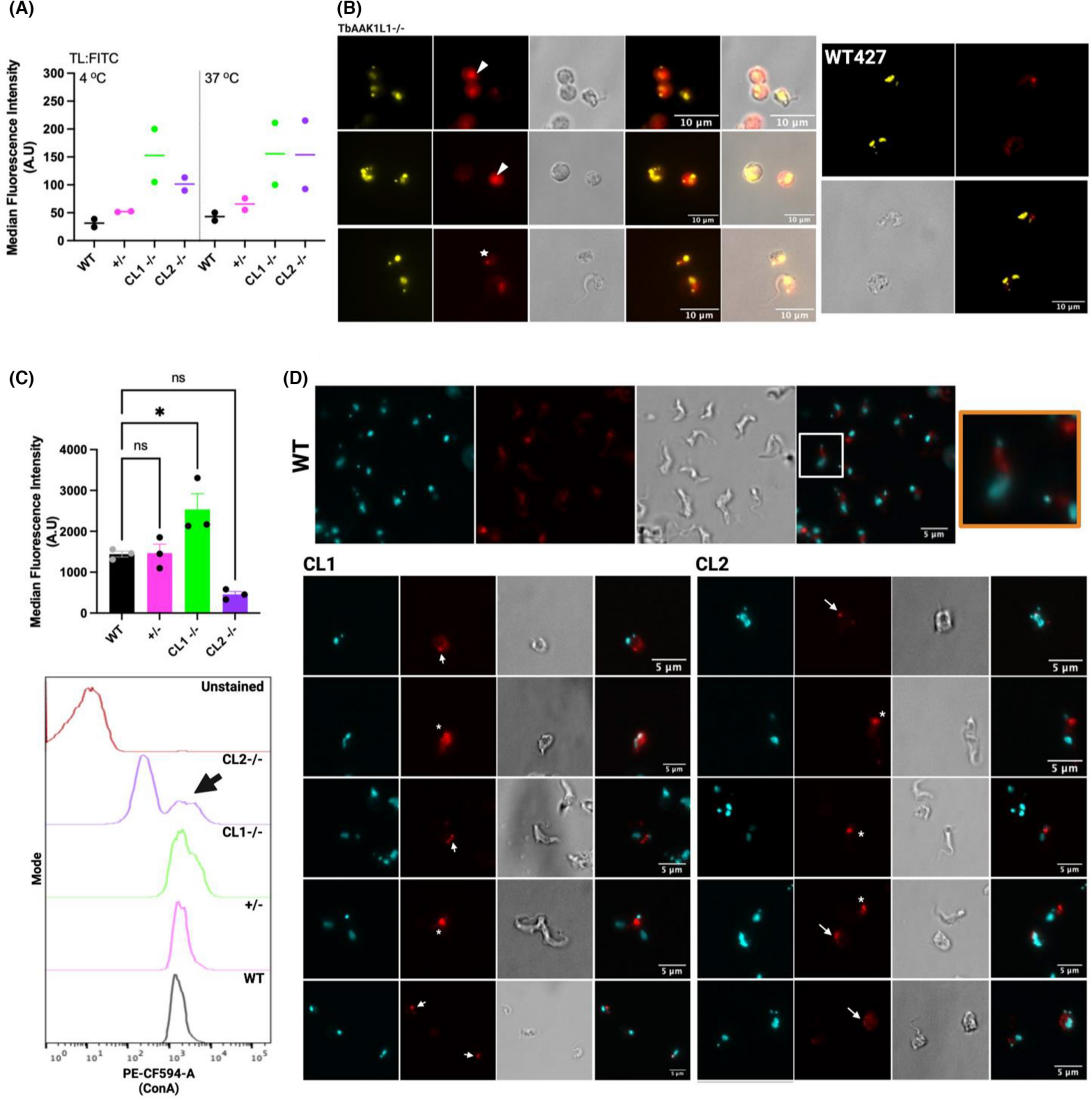
Loss of TbAAK1L1 is associated with impaired cargo uptake. (A) Median Fluorescence Intensity (MFI) was calculated from flow cytometry analysis of TL:FITC binding at 4 and 37°C associated with WT, *TbAAK1L1*+/− cells and two *TbAAK1L1−/−* clones (CL1 and CL2). Dead cells were excluded from this analysis. n = 2 independent experiments, Line = median. (B) Representative images of *TbAAK1L1−/−* CL1 (left) and WT BSF cells (right) showing uptake of TL:FITC (red) at 4°C. White arrow indicates TL in the FP and the white star indicates a rounded cell with no clear enlargement of the FP. Nuclear and kDNA are stained with DAPI and shown in yellow. Images were captured on an Axioskop2 and processed in Image J. Scale bar = 10 μm. Additional images of WT, CL1 and CL2 cells are provided in Data S1. (C) MFI was calculated from the flow cytometry analysis of 594‐conjugated ConA uptake at 37°C associated with WT, *TbAAK1L1*+/− cells and two *TbAAK1L1−/−* clones (CL1 and CL2). Dead cells were excluded from this analysis. Graph shows the MFI of live cells for each cell line. Statistical significance was calculated to compare the MFI of live WT cells with MFIs of heterozygote cells and *TbAAK1L1−/−* clones using a one‐way ANOVA. Error bars SEM. (*) = *p* < 0.05, *n* = 3. Below: Representative flow histogram plot for unstained (brown), WT (black), *TbAAK1L1*+/− (pink), and both *TbAAK1L1−/−* clones (green; CL1, purple; CL2). Plot reflects ConA signal. Black arrow = a population of CL2 cells with increased MFI of ConA. (D) Top Panel: Representative images of 594‐conjugated ConA (red) uptake in control WT cells. An enlarged region is shown in the final orange box panel from the merged image. In all cases DAPI was used to visualize n‐ and kDNA (cyan), ConA is shown in red and differential interference contrast (DIC) was used to image cell morphology. Scale bar = 5 μm. Merged images (far right panels) were generated by merging the DAPI and ConA images. Images were captured on an Axioskop2 (Zeiss). Lower Panels: Representative images of 594‐conjugated ConA (red) uptake from *TbAAK1L1−/−* CL1 (left) and CL2 (right) cells. In all cases DAPI was used to visualize n‐ and kDNA (cyan), ConA is shown in red and differential interference contrast (DIC) was used to image cell morphology. Scale bar = 5 μm. Merged images (far right panels) were generated by merging the DAPI and ConA images. Images were captured on an Axioskop2 (Zeiss). Scale bar = 5 μm. White arrow = indicates a distinct focus of ConA signal. White asterisk (*) = indicates a diffuse focus of ConA signal.

Loss of TbAAK1L1 is associated with FP enlargement, suggesting endocytosis is impaired in these cells. We therefore monitored the binding and uptake of FITC conjugated Tomato Lectin (TL:FITC) at 4 and at 37°C in *TbAAK1L1−/−* cells. In wildtype cells, at 4°C, cargo remains bound within the FP, whereas at 37°C, both binding and uptake of TL:FITC can occur (Nolan et al., 1999). We performed these experiments using flow cytometry on living cells to exclude potential artifacts relating to cell death (Figure 6A and Figure S7A–C). At both temperatures, no significant differences were found between the Median Fluorescent Intensity (MFI) of TL:FITC in WT and *TbAAK1L1+/*− cells (Figure 6A). However, though non‐significant, we observed that loss of *TbAAK1L1−/−* correlated with an increase in the MFI of TL:FITC under both conditions, with no additional accumulation of TL:FITC at 37°C, suggesting TL:FITC was accumulating within the FP and subsequent cargo internalization is hindered. We confirmed this observation by IFA at 4°C (Figure 6B, Figure S7D and Data S1), detecting enlarged FPs positive for TL:FITC signal (white arrow; Figure 6B). We performed a second cargo internalization at 37°C by flow cytometry using concanavalin A (ConA). ConA is another lectin with specificity to mannose and capable of binding glycoproteins on the surface (i.e., representative of surface‐bound cargo; Allen et al., 2003; Schichler et al., 2022). This assay was performed on live cells to exclude cell death‐related artifacts (Figure 6C,D and Figure S7E–G). We found no significant difference between the MFI of ConA signal from WT and heterozygote cell lines (Figure 6C and Figure S7F). In contrast, for *TbAAK1L1−/−* CL1 cells, we found a significant increase in the MFI compared to WT cells (Figure 5A and Figure S7F) suggesting increased ConA uptake in the absence of TbAAK1L1. Unexpectedly, however, we detected a decrease in the MFI of ConA in *TbAAK1L1−/−* CL2 cells, contradicting the results for obtained using CL1. However, when we examined the cytometry profile in more detail, we observed the emergence of two apparently distinct populations, with ~60% of CL2 cells appearing unstained, or ‘weakly’ stained for ConA (Figure 6C; Data S1). The remaining ~40% of parasites appeared to have increased ConA fluorescence (this population is indicated by a black arrow in Figure 6C). When the latter population was isolated by gating, a significant increase in the MFI of ConA for CL2 was observed, like CL1 (Data S2). We are unable to account for these differences between CL1 and CL2 as we found no other observable differences between the two clones, despite this effect being consistent across replicates (discussed in more detail below). We next performed immunofluorescence to localize ConA in fixed parasites. DAPI was used to visualize the n‐and kDNA. As expected, ConA became internalized in WT and heterozygote control cells (Figure 6D; Figure S7G), appearing as a diffuse, cytoplasmic signal between the kDNA and the nuclear DNA. In contrast, for both *TbAAK1L1−/−* clones we observed bright, enlarged concentrations of ConA signal appearing to accumulate within, or in close proximity, to the FP (Figure 6D). In some cases, the foci appeared at a distance from the kDNA, though it is possible the morphological aberrancies may disrupt the location of ConA accumulation. No clear differences between CL1 and CL2 were visually detected.

Overall, despite the differences between our two null mutant cell lines, these ConA data, taken together with the TL:FITC data, suggest TbAAKL1 loss correlates with impeded entry of surface bound cargo into BSF *T. brucei*. We suggest this explanation is more likely than loss of TbAAK1L1 altering fluid phase entry, since we see enlarged FPs in the mutants, though wider alterations cannot be discounted. Indeed, it is important to note that we have not tested directly for alterations to fluid phase entry in this study.

## DISCUSSION

By exploring the genomes of a range of available eukaryotes, we reveal surprising complexity in the evolution of AAK1. First, we propose AAK1 duplication in the kinetoplastid ancestor resulted in two paralogues, AAK1L1 and AAK1L2. Expansion of AAK1 gene number may not be unique to this protozoan grouping, since more limited species sampling of *Trichomonas* indicates the potential for multiple paralogues (Figure S1). Second, within the Kinetoplastida we find evidence of further evolutionary diversification, with complete loss of AAK1L2 in all sampled salivarian trypanosomes and unique evolutionary selection on AAK1L1 in the same trypanosomes (Figure 1). The non‐synonymous replacement of important functional residues within the P‐loop, HRD and DFG kinase motifs in AAK1L1 orthologues suggests these proteins are pseudokinases in salivarian trypanosomes. This predicated lack of kinase activity may not be limited to salivarian trypanosome species, since replacement of functional residues was seen in non‐salivarian trypanosomes, though more detailed sequences analyses are required in individual species. In other kinetoplastids, including *Leishmania* (Figure 2), AAK1L1 orthologues appear functional. In contrast, AAK1L2 may be a functional kinase across the kinetoplastids. Absence of AAK1L2 and likely loss of kinase function in AAK1L1 correlates with observed lack of all AP‐2 complex subunits across the salivarian trypanosomes and coincides with acquisition of the VSG coat (Manna et al., 2013). Curiously, a DPF motif involved in interaction of AAK1 with the AP‐2 complex (Neveu et al., 2015; Olusanya et al., 2001) was found within the C‐terminal region of all AAK1L1, but not AAK1L2, orthologues. Unlike in HsAAK1, however, a second DPF motif, or a WNPF (conforming to a WXX(F/W) pattern) motif, which has also been shown to bind AP‐2α (Jha et al., 2004; Mishra et al., 2004), was not identified. What form of AAK1L1 interaction, if any, is mediated by a single DPF motif is unknown. The AP‐2 complex is found in both *Leishmania* sp. and non‐salivarian trypanosomes but remains uncharacterised. It would be interesting to investigate whether AAK1 homologues bind AP‐2 complex components in these lineages. The implications of AAK1L1 orthologues potentially lacking kinase activity in at least some non‐salivarian trypanosomes is also worth exploring. Our sequence analyses suggest salivarian AAK1L1 proteins likely underwent a period of positive selection in a subset of positions concurrent with AP‐2 loss, but are predominantly constrained under negative selection, suggesting that they retain functional significance; a prediction we confirm in *T. brucei*.

TbAAK1L1 interacts with clathrin (Manna et al., 2017) and localizes in proximity to the FP. We confirm TbAAK1L1 localizes between the kDNA and the nDNA, where other components of the polarized endosome system are also found in *T. brucei* (Broster Reix et al., 2021; Field & Carrington, 2009; Florimond et al., 2015; Halliday et al., 2021; Manna et al., 2017; Morriswood & Schmidt, 2015; Perdomo et al., 2022; Schichler et al., 2022). Moreover, we demonstrate that TbAAK1L1 can be found in contact with the FP membrane and endocytic vesicles, suggestive of a role for TbAAK1L1 during cargo internalization. By localizing within this region, the protein has access to the FP membrane, potentially incoming vesicles, and the flagellum. If the protein is indeed a pseudokinase, it might be predicted to provide a structural and/or other role as reported for other pseudokinases in other organisms (Mace & Murphy, 2021; Putters et al., 2011; Tomoni et al., 2019) and therefore identifying what factors, if any, it binds (beyond clathrin) may be revealing. TbAAK1L1 was described as a putative interaction partner of the Tip of the Extending FAZ1 protein (TOEFAZ1/the cytokinetic initiation factor 1; CIF1) by proximity‐dependent biotinylation (BioID; Hilton et al., 2018; Sinclair‐Davis et al., 2017). TOEFAZ1 is a putative interaction partner and regulator of the polo‐like kinase (TbPLK), a cytokinesis regulator, by aiding TbPLK recruitment to the tip of the newly forming attachment zone for the flagellum (the flagellar attachment zone; FAZ). TbAAK1L1 was not further characterized in this study, but such data could indicate TbAAK1L1 plays roles beyond a putative endocytic function, particularly as TOEFAZ1 was not identified as a clathrin interacting protein. However, in another study performed to identify the interactors of CIF1/TOEFAZ (Zhou et al., 2016, 2018), TbAAK1L1 was not identified. Indeed, we observed no clear evidence of cytokinetic defects in the null mutants we generated. The dynamic, cell cycle associated localization of TbAAK1L1 we describe likely reflects events occurring during the establishment of a new, daughter FP. In this regard, the appearance of a second fluorescent TbAAK1L1 focus coincides with the timing of kDNA replication and division, at which point the FPs of the two cells begins to separate laterally (Wheeler et al., 2019).

We (Stortz et al., 2017), and others (Jones et al., 2014) have now shown independently, by RNAi and the generation of null mutant cells (this study), that TbAAK1L1 loss leads to proliferative defects and the formation of cells consistent with defective endocytosis. Deletion of TbAAK1L1 correlates with a gross enlargement of the FP (Figure 5) and increased TL and ConA signal (Figure 6), coinciding with the accumulation of both TL and ConA within the FP. Endocytosis defects similar to those reported here, including blocked or slowed ConA uptake, have been previously reported following the depletion of clathrin, TbMORN1, TbSmee1 and other FPC/HC members in *T. brucei* (Allen et al., 2003; Broster Reix et al., 2021; Florimond et al., 2015; Halliday et al., 2021; Hung et al., 2004; Morriswood & Schmidt, 2015; Perdomo et al., 2022; Schichler et al., 2022). RNAi and null mutant cell lines exhibit similar morphological defects (Figure 4C,D), suggesting that the aberrant cells observed in the null mutant cell lines are unlikely a consequence of long‐term culture adaptation. The discrepancy in extent of observed effects following TbAAK1L1 loss in these published studies is unclear. A further discrepancy reported here pertains to differences in ConA uptake between the two independently generated null mutant clones. Nonetheless, we describe ConA appearing to accumulate within the FP of both null mutant clones, suggesting internalization of cargo is impaired in TbAAK1L1's absence. Moreover, altered uptake of TL:FITC at 37°C (assayed by microscopy and flow cytometry) further supports a blockage in cargo internalization in the mutants and provides further evidence of a role for TbAAK1L1 in *T. brucei* endocytosis. Why specifically ConA uptake is impaired in most of the population of only mutant CL2 cells is unclear. However, in comparison to TL, ConA is a larger (~104 kDA unconjugated) tetrameric molecule (TL is monomeric and 71 kDa unconjugated). Perhaps, therefore, a possible explanation for the clone discrepancy is that the rate of endocytosis is slower in CL2 relative to CL1, meaning a large, complex molecule like ConA may require additional time in this particular mutant to accumulate in the FP. However, direct measurement of endocytosis rate would be needed to address this question.

Two models of cargo sorting for internalization prior to uptake into the flagellar pocket have been proposed: the size exclusion, and the membrane subdomain models. The size exclusion model suggests that factors including TbMORN1 and TbSmee1 function to structurally support the flagellar pocket neck (FPN), allowing access for macromolecules (like ConA; or ‘surface‐cargo’) to the pocket. The membrane subdomain model proposes that a region of membrane is associated with ConA binding and TbMORN1 facilitates the internalization of this membrane region. Broadly, current data favors the former (Morriswood & Schmidt, 2015). The lack of the AP‐2 complex in *T. brucei* largely suggests CME is not required during the initial sorting of incoming cargo, but rather sorting occurs post‐internalization. Our data here suggests that TbAAK1L1 is not required for FP binding but instead may operate at an initial point of entry for certain cargo species into the parasite. However, TbAA1KL1 mutants are notable for the presence of internal vesicle‐like structures, meaning we cannot definitively rule out additional sorting functions for TbAAK1L1. In this case, we assume that TbAAK1L1 acts separately from AP‐2‐independent CME, suggesting a distinct entry pathway into the trypanosome cell operates. However, TbAAK1L1 does interact with clathrin (Manna et al., 2017), so a role in a variant form of CME, uncoupled from AP‐2, remains possible. Why such a role for the putative pseudokinase is non‐essential is unclear. It is important to stress, however, that any putative endocytosis impediment is partial, since not all TbAAK1L1 mutant cells accumulate the defects we describe, and the null mutants are viable, at least under in vitro culture conditions. We did not directly test for alterations to fluid‐phase cargo dynamics; thus, we are unable to conclude if loss of TbAAK1L1 is required for that process. Given TbAAK1L1‐clathrin interaction, it seems likely the loss of TbAAK1L1, like the loss of clathrin, does not hinder fluid cargo entering the pocket, consistent with the enlarged FP in these parasites. Indeed, with a high rate of the VSG coat turnover, the lack of AP‐2 may be beneficial (Manna et al., 2013), allowing rapid VSG coat internalization. Other surface coat factors are instead ‘sorted’ in a receptor‐mediated manner, which is directed by RME‐8 (Koumandou et al., 2013). It is unknown whether other proteins use the RME‐8 signal to facilitate initial uptake into the cell.

In summary, we reveal a role for a putative pseudokinase during *T. brucei* BSF endocytosis. Furthermore, the identification of the paralogous TbAAK1L2 supports divergence of CME, cargo selection, and sorting in this eukaryotic parasite relative to other kinetoplastid parasites. Importantly, and despite apparent divergence in the endocytic pathway compared to most eukaryotes, TbAAK1L1 at least partially retains an endocytic function seen in other AAK1 homologues, as has been suggested for other proteins involved in trafficking (Klinger et al., 2016). These insights may also have wider significance for eukaryotic biology since endocytosis across synaptic vesicles in other cells also does not require an AP‐2 complex (Gu et al., 2008). Furthermore, the involvement of pseudokinases in an endocytosis pathway is not unprecedented: in human cells, for instance, the Janus kinase, JAK2 (via its pseudokinase domain), can perform endocytic functions independent from its kinase activity (Putters et al., 2011).

## AUTHOR CONTRIBUTIONS

JAB, JCM, and RM conceived the study; JAB, CMK, LL, and RM performed the research and/or analyzed the data; JAB, CMK, LL, JCM, JBD, and RM wrote and edited the manuscript.

## CONFLICT OF INTEREST STATEMENT

All the authors: no conflicts reported.

## Supporting information


**Figure S1.** Phylogenetic analysis of Nek‐like and numb‐associated kinase (NAK) homologues.
**Figure S2.** Selective pressure on AAK1L1 orthologues.
**Figure S3.** Endogenous tagging of TbAAK1L1.
**Figure S4.** TbAAK1L1 subcellular localisation by ImmunoGold® labelling.
**Figure S5.** *TbAAK1L1−/−* null mutant strategy and confirmation of mutants.
**Figure S6.** Loss of TbAAK1L1 is associated with alterations to the internal architecture of BSF cells.
**Figure S7.** Measurements of ConA uptake in the absence or depletion of TbAAK1L1 using flow cytometry.
**Data S1.** Additional images of TL:FITC binding in wild type and null mutant cell lines.
**Data S2.** Flow cytometry analysis of ConA uptake at 37°C.

**Table S1.** Homology analysis of kinetoplastid NAK‐like protein kinases.

**Table S2.** Analysis of selection pressure acting on kinetoplastid AAK1‐like protein kinases.
