# Supplementary material for: AAK1‐like: A putative pseudokinase with potential roles in cargo uptake in bloodstream form Trypanosoma brucei parasites
Source: J Eukaryot Microbiol. 2023 Aug 7;70(6):e12994. doi: 10.1111/jeu.12994 (PMC10952953; doi:10.1111/jeu.12994)
Supplement: Supplementary file 1 — Figure S1. Phylogenetic analysis of Nek‐like and numb‐associated kinase (NAK) homologues. Figure S2. Selective pressure on AAK1L1 orthologues. Figure S3. Endogenous tagging of TbAAK1L1. Figure S4. TbAAK1L1 subcellular localisation by ImmunoGold® labelling. Figure S5. TbAAK1L1−/− null mutant strategy and confirmation of mutants. Figure S6. Loss of TbAAK1L1 is associated with alterations to the internal architecture of BSF cells. Figure S7. Measurements of ConA uptake in the absence or depletion of TbAAK1L1 using flow cytometry. Data S1. Additional images of TL:FITC binding in wild type and null mutant cell lines. Data S2. Flow cytometry analysis of ConA uptake at 37°C. [file JEU-70-0-s003.pdf]

SUPPORTING INFORMATION

AAK1-like: a putative pseudokinase with potential roles in cargo uptake in bloodstream form *Trypanosoma brucei* parasites by Jennifer A. Black, Christen M. Klinger, Leandro Lemgruber, Joel B. Dacks, Jeremy C. Mottram and Richard McCulloch

Figure S1. Phylogenetic analysis of Nek-like and NAK homologues.
This figure depicts a clear split between NIMA/Nek-like kinases, including Tb927.10.460, and NAK family kinases, including Tb927.910.6560. The best maximum-likelihood topology is shown, together with bootstrap support. Support for all internal nodes is denoted by symbols as per figure inset, whereby the support value is at least that shown for the relevant symbol.

Figure S2. Selective pressure on AAK1L1 orthologues.
(A) Schematic illustration of conserved motifs and domains in human AAK1 and *T. brucei* AAK1L1. The polypeptide sequences of *H. sapiens* AAK1 and *T. brucei* AAK1L1 (TbAAK1L1) are shown in purple; predicted numbers of amino acids (aa) indicated (not to scale). Conserved sequence motifs of AAK1 are annotated as dotted triangles and coloured blocks, while the larger kinase domain is shown as a red block. Positions of the domain and motifs, and their potential conservation in TbAAK1L1, were determined using Interpro (http://www.ebi.ac.uk/interpro/). (B) Position-specific dN/dS values for AAK1L1 orthologues are shown, as inferred using SLAC. Graphs are shown for all orthologues (top), salivarian trypanosomes only (lower right), and all taxa other than salivarian trypanosomes (lower left). (C) Bokeh Plot generated using IUPred2A (https://iupred2a.elte.hu) from the amino acid sequence of TbAAK1L1 and AAK1 (accession numbers as stated in A). The predicted location of the kinase domain is shown. A large proportion of the C-termini of both factors is disordered (red line). The blue line (ANCHOR score) reflects putative binding sites within regions of protein disorder that could reflect putative sites of interactions with other factors. (D) Modelling predictions (*in silico*) of the structures of the human AAK1 and TbAAK1L1 proteins using Phyre2 (-intensive mode-)86. Protein structures are coloured from N-terminus (red) to C-terminus (blue). Due to the disordered nature of both proteins, less than 35% of each protein could be modelled with confidence (100% confidence). Over 60% of the protein predicted to be disordered. Accession numbers for each protein sequence are as state in (A).

Figure S3. Endogenous tagging of TbAAK1L1.
(A) Schematic diagram of endogenous tagging strategy. Black arrows indicate approximate primer localisation in the genome, showing one endogenous ORF translationally fused to 12myc (top) and the second allele (bottom) disrupted by integration of an antibiotic resistance cassette (G418 flanked by tubulin and actin intergenic sequences). Diagram not to scale. (Right side) PCR reactions testing integration of the G418 ORF replacing the TbAAK1L1 ORF for one allele. All PCRs were performed on the same gDNA and ddH20 was used as a negative control. B/α tub (Beta/Alpha Tubulin), Actin IR (Actin intergenic region), WT (Wild type), gDNA (genomic DNA), ddH_20 (double distilled water). UTR (untranslated region), ORF (open reading frame). Sizes shown (kb plus ladder; bp). (Left side) No proliferative defects were observed when growth of TbAAK1L1$^{+/-12myc}$ cells were compared relative to the growth of WT 427 cells and untagged *TbAAK1L1+/-* cells. Error bars ± SEM; n = 3. (B) Representative images of the subcellular localisation of TbAAK1L1$^{+/-12myc}$ in BSF cells throughout the cell cycle. Indirect

immunofluorescence was performed using anti-myc antiserum conjugated to FITC (shown in red). DAPI was used to visualise the n- and kDNA (shown in grey). Images were captured on an Axioskope2 (Zeiss). Scale bar = 5 µm. Lower panel: Representative image of WT untagged cells used as a control for non-specific binding of the anti-myc antiserum. Anti-myc antiserum conjugated to FITC (shown in red) with DAPI used to visualise the n- and kDNA (shown in grey). Scale bar = 10 µm. White arrow indicates a second focus of myc signal in TbAAK1L1$^{+/-12myc}$ cells (C) Representative images of the subcellular localisation of TbAAK1L1$^{+/-12myc}$ in BSF cells after cytoskeletal extractions were performed using NP-40. WT cells are used as a control. No anti-myc localisation could be determined following cytoskeletal extractions. Indirection immunofluorescence was performed using anti-myc conjugate to Alexa Fluor 488 (red) and the n- and k-DNA stained with DAPI (shown in grey). Cytoskeletons were imaged using DIC microscopy. Scale bar = 10 µm. Images were captured on an Axioskope2 (Zeiss) and processed in Image J.

Figure S4. TbAAK1L1 subcellular localisation by ImmunoGold® labelling
Representative images of WT cells (top panel) as a control for non-specific antibody binding. Scale bars are as shown on each image. Lower Panel: Additional representative images or the uncropped versions of images presented in Figure 3 F of the subcellular TbAAK1L1 localisation by ImmunoGold® staining. 10 nm gold particles were used for these experiments. Scale bars are as annotated on each image. FP = Flagellar Pocket, White arrow = a gold particle in contact with a vesicle. Images were captured on a Tecnai T20 EM microscope and processed in ImageJ.

Figure S5. *TbAAK1L1-/-* null mutant strategy and confirmation of mutants.
(A) Schematic diagram of the strategy to generate *TbAAK1L1-/-* null mutants. Black arrows indicate approximate regions amplified by PCR to confirm mutant cell lines. Fragment sizes are shown above the arrows and in brackets; the primer numbers required for amplification of the region are shown. Diagram not to scale. (B) Agarose gels of PCRs using the primers detailed in (A). PCR was used to confirm integration in the appropriate genomic locus of both the G418 (referred to also as NEO) and BSD resistance cassettes. gDNA from two -/- clones (CL1 and CL2), WT 427 cells and *TbAAK1L1+/-* cells was used. ddH$_2$0 was used as a negative control. All PCRs were performed on the same gDNA samples. Size markers are shown (bp). BSD (Blasticidin), NEO (neomycin/G418), B/α tub (Beta/Alpha Tubulin), Actin IR (Actin intergenic region), gDNA (genomic DNA). (C) Cell cycle analysis of WT, *TbAAK1L1-/-* and *TbAAK1L1+/-* cells *in vitro*. DAPI was used to visualise the n- and kDNA. The number of 1N1K, 1N2K, 2N2K and 'other' cells were counted and expressed as a percentage of the total population (over 200 cells per cell line were counted per experiment). Error bars (± SEM); n = 3 independent experiments; (****) = $p < 0.0001$. Statistical significance was calculated to compare WT cell cycle population with the corresponding *TbAAK1L1-/-* clone (CL1 and CL2) densities using a one-way ANOVA. Upper Panel: Representative images of n- and k-DNA configurations in WT cells used to categorise cells are shown. Cells were stained with DAPI (to visualise n- and kDNA; shown in grey) and α KMX-1 (to visualise β tubulin; shown in red). Images were captured on an Axioskop2 (Zeiss). Scale bar = 5 µm. representative images of *TbAAK1L1-/-* 'other' cells (from CL1). Cells were stained with DAPI (to visualise n- and kDNA; shown in grey) and α KMX-1 (to visualise β tubulin; shown in red). Images were captured on an Axioskop2 (Zeiss) and processed in Image J. Scale bar = 5 µm. (D) Field of view images of WT, *TbAAK1L1+/-* and both *TbAAK1L1-/-* clones. Cells were stained with DAPI (to visualise n- and

kDNA; grey), anti-KMX1 (to visualise tublin; red) and the cell body imaged using DIC. Scale bar = 10 µm. Images were captured on an Axiskop2 (Zeiss) and processed in ImageJ.

Figure S6. Loss of TbAAK1L1 is associated with alterations to the internal architecture of BSF cells
(A) Examples of Wild type (WT 427) and *TbAAK1L1+/-* cells were fixed and imaged using a Tecnai T20 transmission electron microscope. Scale bar = 1 µm and 0.5 µm. (B) Table shows scoring across of the cells captured by TEM on the appearance or absence of an enlarged FP. (C) Images show electron dense material collecting in vesicles (left; indicated by a white arrow) or within the FP (right; indicated by a black arrow) in *TbAAK1L1-/-* CL1 cells. (D) Images show the point at which the flagellum enters the FP (via the FP collar). Enlarged sections (indicated by a black box) are shown below the appropriate image. Scale bars = 0.5 µm. FP = flagellar pocket.

Figure S7. Measurements of ConA uptake in the absence or depletion of TbAAK1L1 using flow cytometry.
Flow cytometry plots showing TL:FITC binding at 4 ºC (A) and 37 ºC (B) for WT, heterozygote, and null mutant cell lines for the two independent biological replicates performed. (C) Representative gating strategy used to investigate the TL:FITC fluorescent signal in WT, heterozygote and *TbAAK1L1-/-* cell lines. Propidium Iodide (PI) positive control cells were generated by heat-treating WT BSF cells at 70 ºC for 3 mins to kill most of the population (>90%). (D) Representative IFA images showing TL:FITC (red) subcellular localisation in *TbAAK1L1+/-* cells at 4 ºC. Cells were fixed and the nuclear and kinetoplast DNA visualised by DAPI staining (yellow). Cell body was imaged by DIC. Scale bar = 10 µm. Images were captured on an Axioskop2 (Zeiss) and processed in ImageJ. (E) Representative gating strategy used to investigate 594-conjugated ConA fluorescent signal in WT, heterozygote and *TbAAK1L1-/-* cell lines. DAPI positive control cells were generated by heat-treating WT BSF cells at 70 ºC for 3 mins to kill most of the population (>90%). (F) Flow cytometry plots showing ConA uptake for WT, heterozygote, and null mutant cell lines for the two other independent biological replicates performed (R2 = Replicate2, R3 = Replicate 3). (G) Representative IFA images showing ConA (red) uptake in *TbAAK1L1+/-* cells after 30 mins at 37ºC. Cells were fixed and the nuclear and kinetoplast DNA visualised by DAPI staining (cyan). White boxes highlight cells seen in the blown-up images on the right of the panel. Scale bar = 5 µm. Images were captured on an Axioskop2 (Zeiss) and processed in ImageJ.

Data S1: Additional images of TL:FITC binding in WT and null mutant cell lines.

Data S2: Flow cytometry analysis of ConA uptake at 37 oC. Cells were instead gated based on ConA fluorescence. The percentage of ConA positive cells across all cell lines (A), the gating strategy (B), the MFI of ConA +ve cells (C; significance is as described in Figure 6C) with the corresponding cytometry plot (upper) and lower), the two other replicates of this experiment (D).

Table S1. Homology analysis of kinetoplastid NAK-like protein kinases.

Table S2. Analysis of selection pressure acting on kinetoplastid AAK1-like protein kinases.

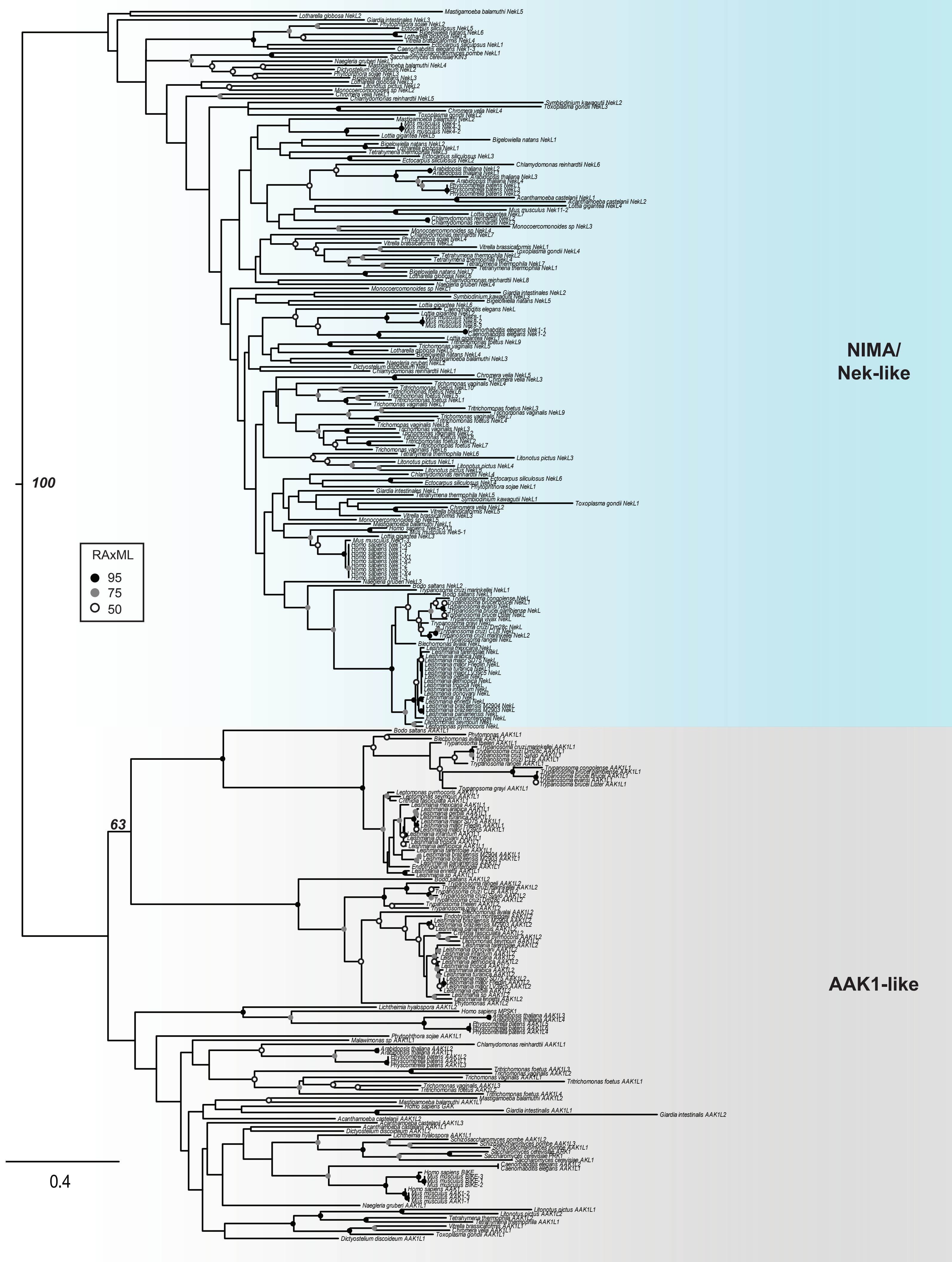
NIMA/
Nek-like
AAK1-like
RAxML
95
75
50
100
63
0.4

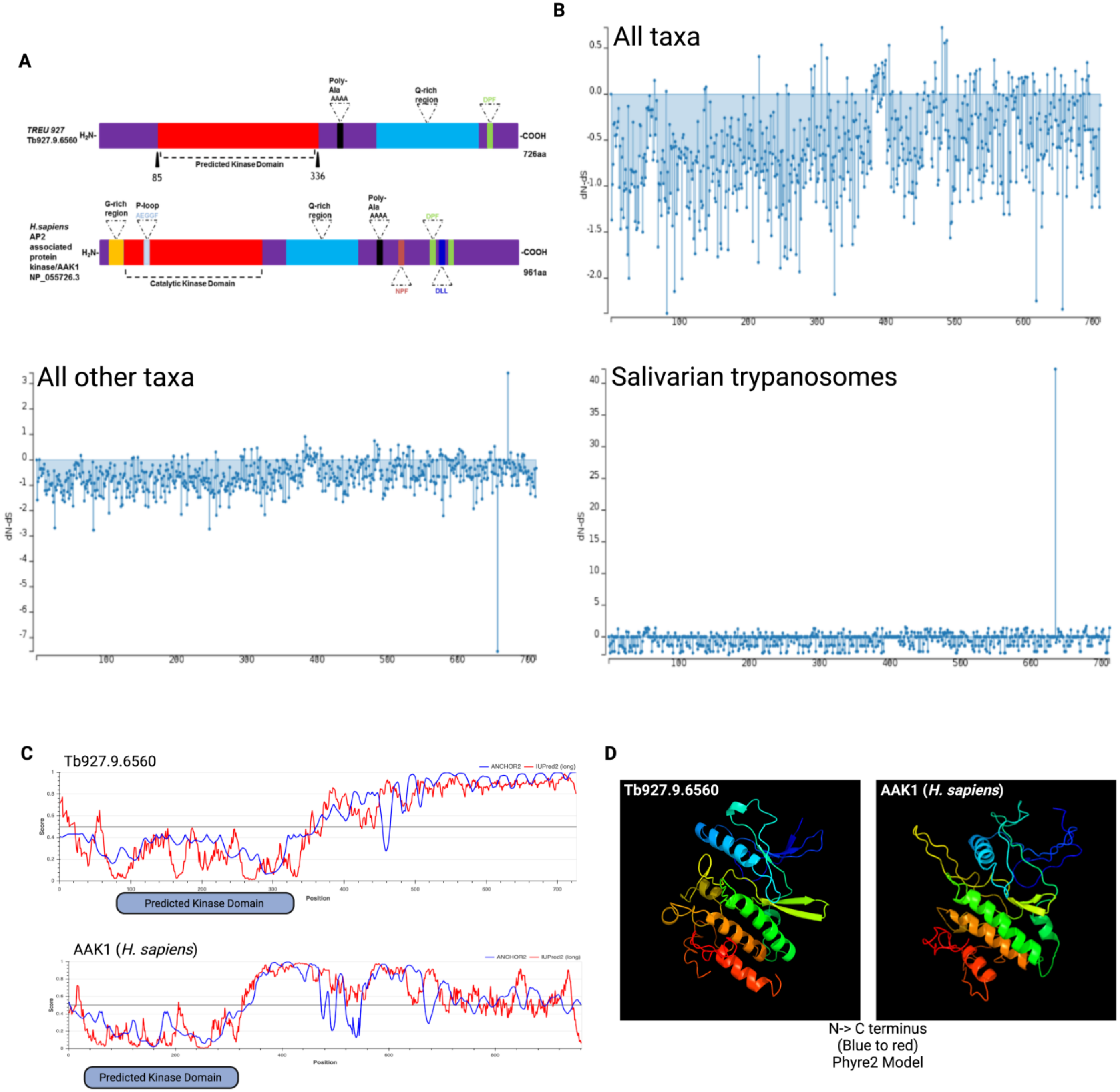
A
TREU 927
Tb927.9.6560
Poly-Ala AAAA
Q-rich region
DPF
-COOH
726aa
Predicted Kinase Domain
85
336
H.sapiens AP2 associated protein kinase/AAK1 NP_055726.3
G-rich region
P-loop
Catalytic Kinase Domain
NPF
DLL
961aa
B
All taxa
All other taxa
Salivarian trypanosomes
dN-dS
C
Tb927.9.6560
ANCHOR2
IUPred2 (long)
Score
Position
Predicted Kinase Domain
AAK1 (H. sapiens)
D
Tb927.9.6560
AAK1 (H. sapiens)
N-> C terminus
(Blue to red)
Phyre2 Model

Figure S2

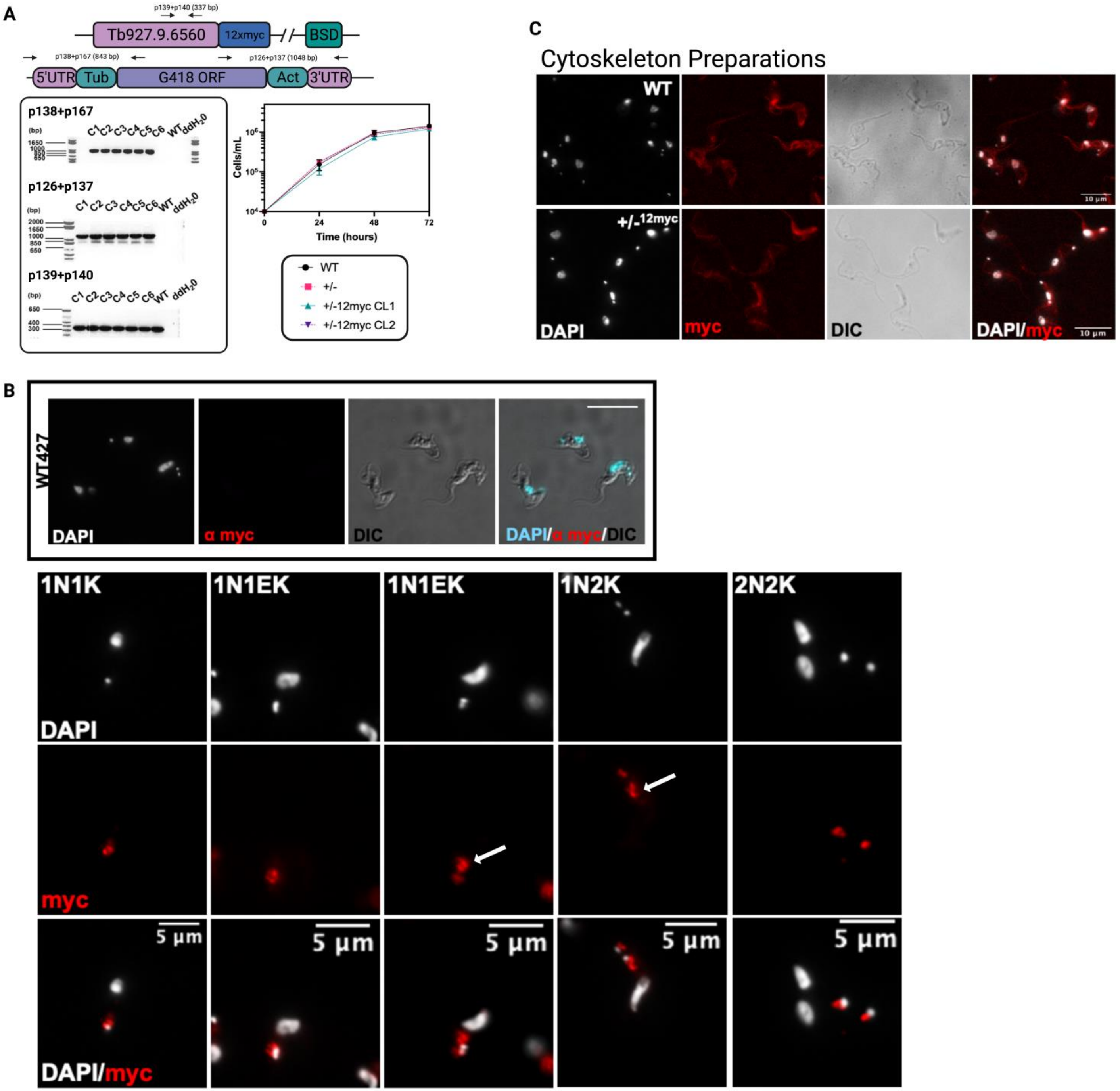

A
p139+p140 (337 bp)
Tb927.9.6560
12xmyc
BSD
p138+p167 (843 bp)
p126+p137 (1048 bp)
5'UTR
Tub
G418 ORF
Act
3'UTR
p138+p167
C1 C2 C3 C4 C5 C6 WT ddH2O
p126+p137
p139+p140
Cells/mL
Time (hours)
WT
+/-
+/-12myc CL1
+/-12myc CL2
C
Cytoskeleton Preparations
WT
+/-12myc
DAPI
myc
DIC
DAPI/myc
10 µm
B
WT427
α myc
DAPI/α myc/DIC
1N1K
1N1EK
1N2K
2N2K
5 µm

Figure S3

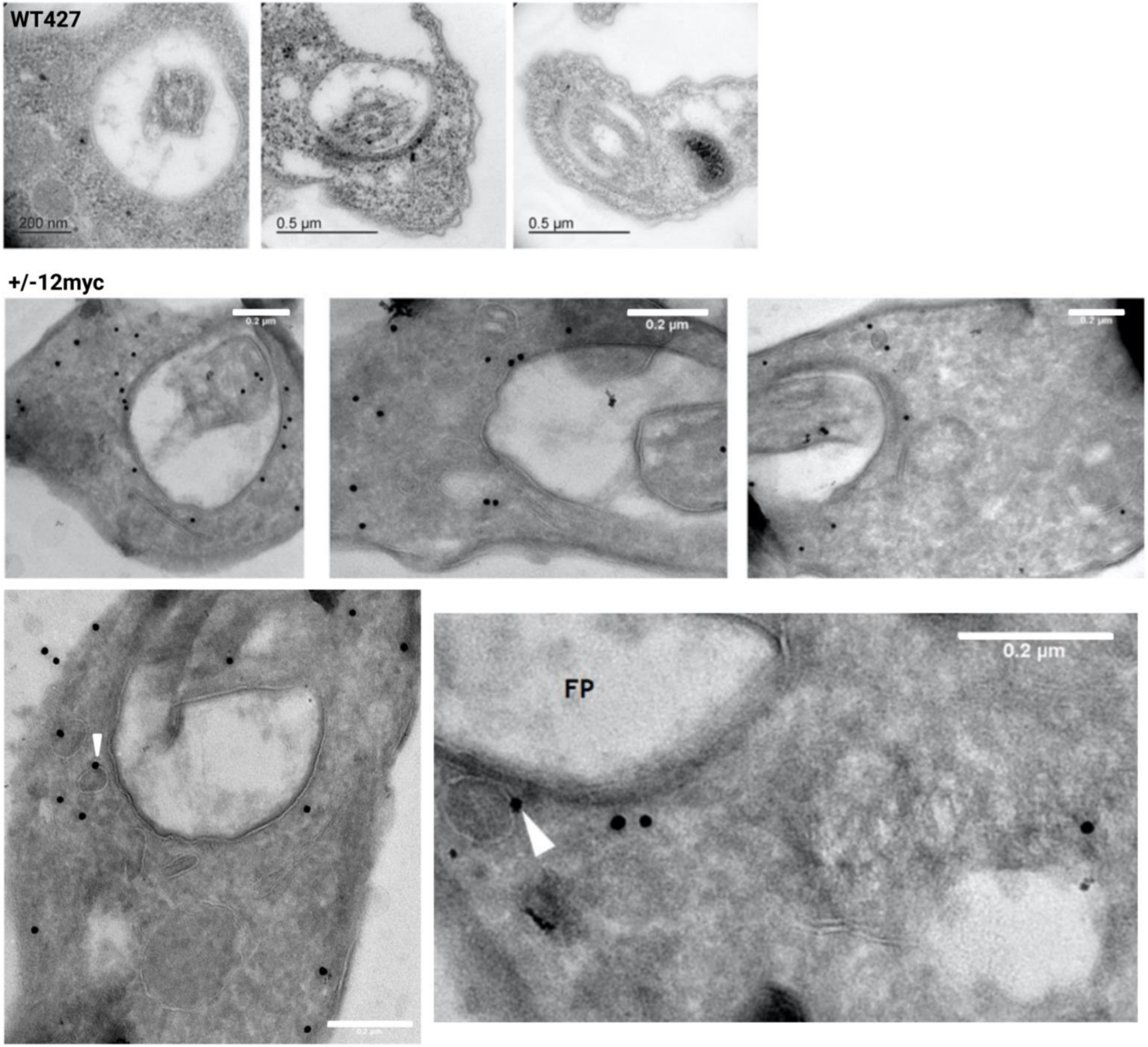

Figure S4

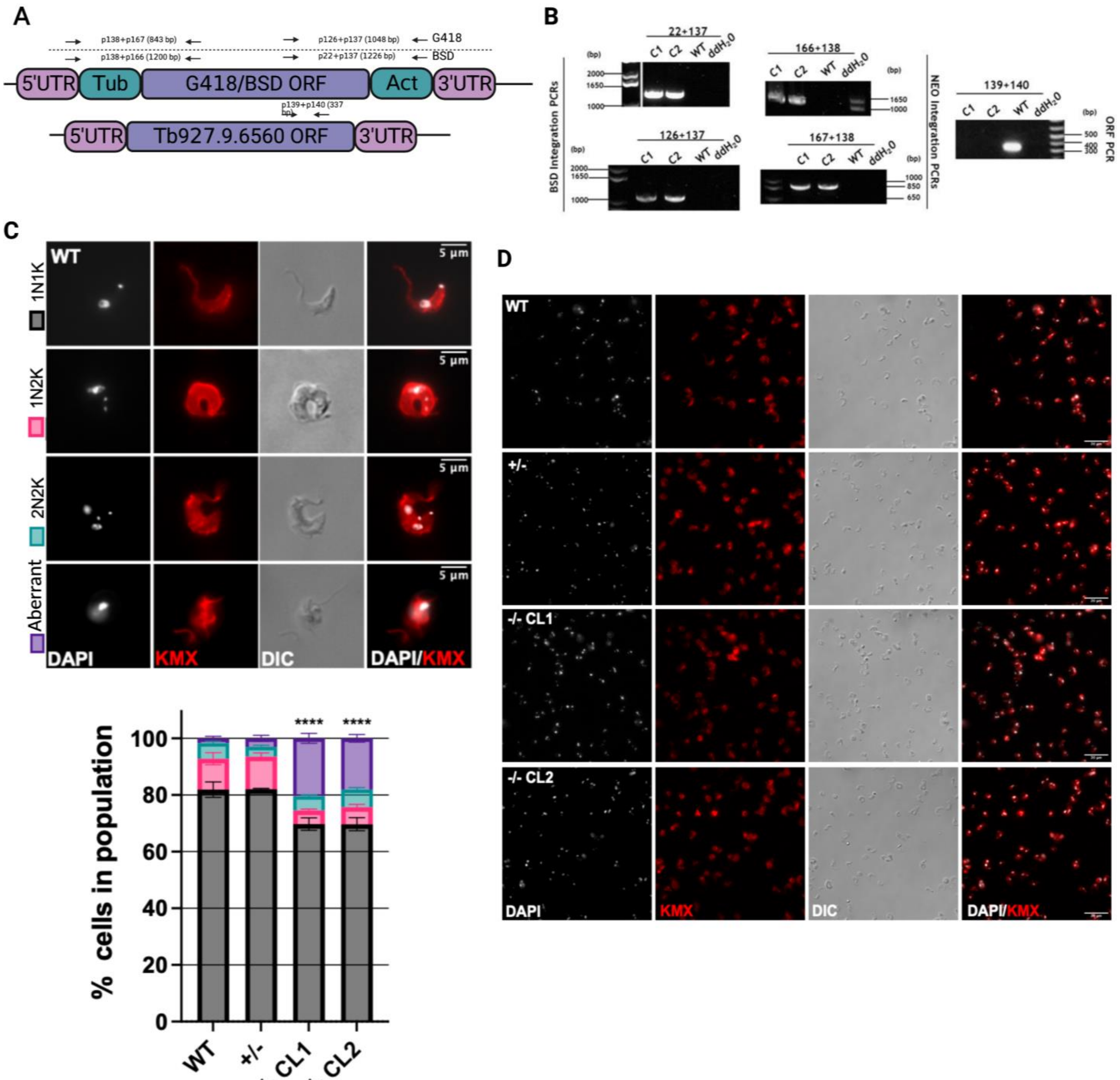
A
p138+p167 (843 bp)
p126+p137 (1048 bp)
G418
p138+p166 (1200 bp)
p22+p137 (1226 bp)
BSD
5'UTR
Tub
G418/BSD ORF
Act
3'UTR
Tb927.9.6560 ORF
B
BSD Integration PCRs
NEO Integration PCRs
ORF PCR
22+137
126+137
166+138
167+138
139+140
C1
C2
WT
C
1N1K
1N2K
2N2K
Aberrant
5 µm
DAPI
KMX
DIC
DAPI/KMX
% cells in population
WT
+/-
-/- CL1
-/- CL2
****
D

Figure S5

A

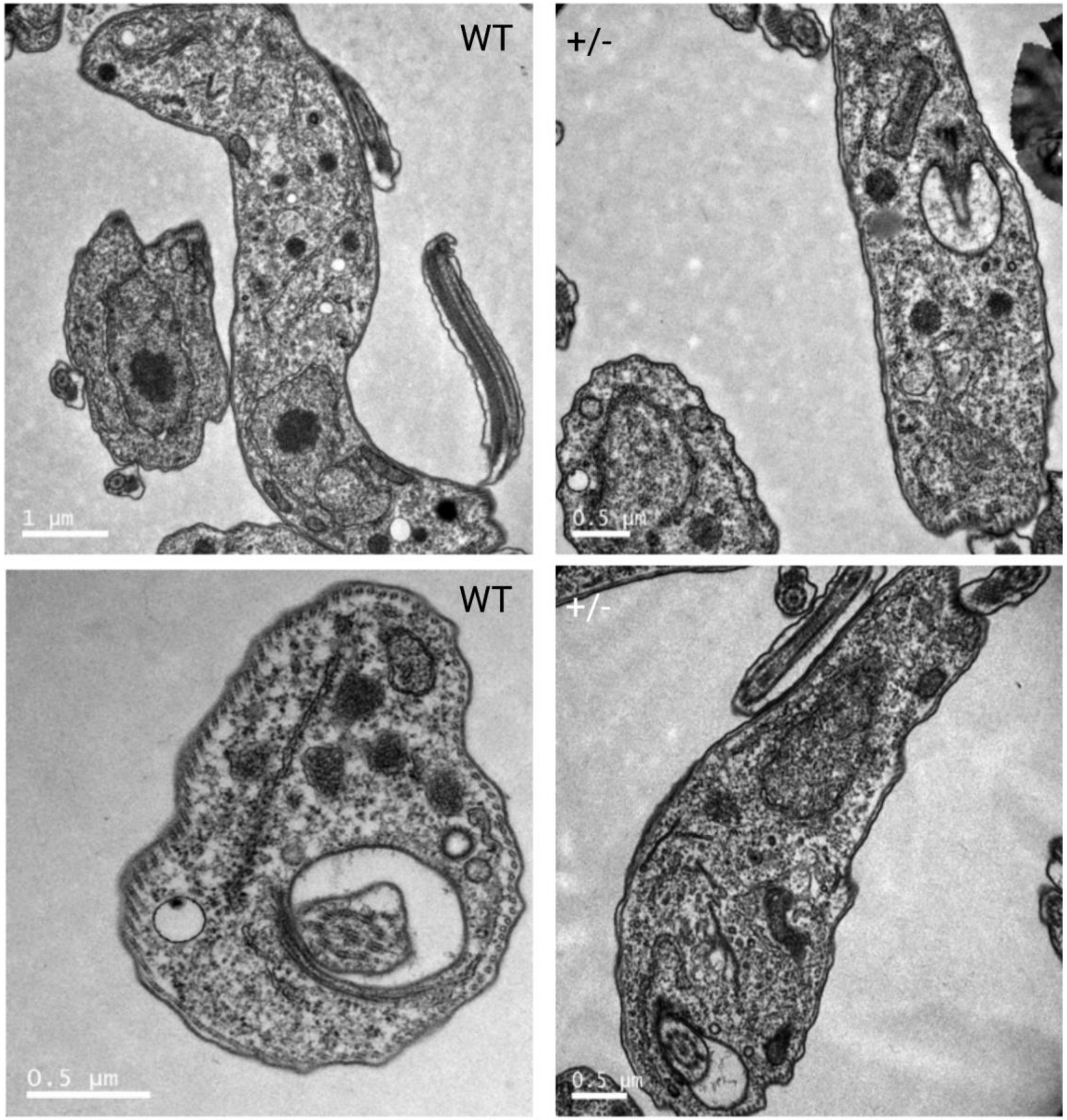

B

Phenotypic Feature	WT (17)	+/- (13)	-/- CL1 (34)
Enlarged FP	0	0	27

C

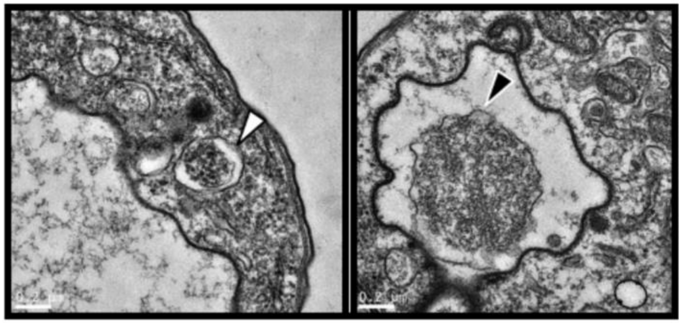

D

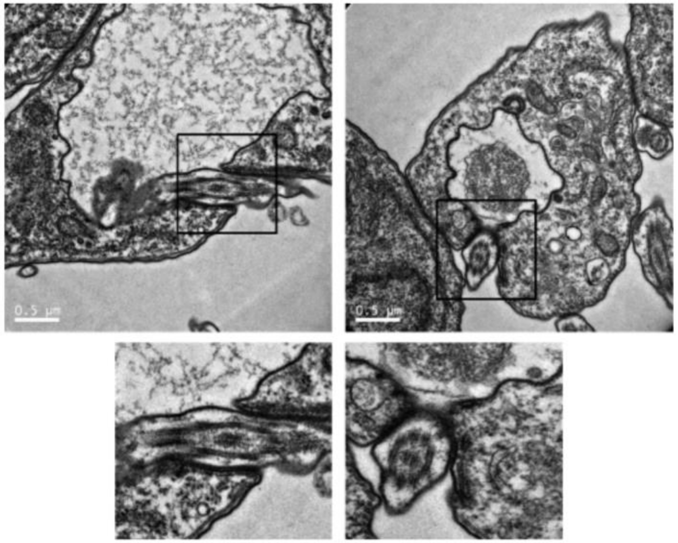

Figure S6

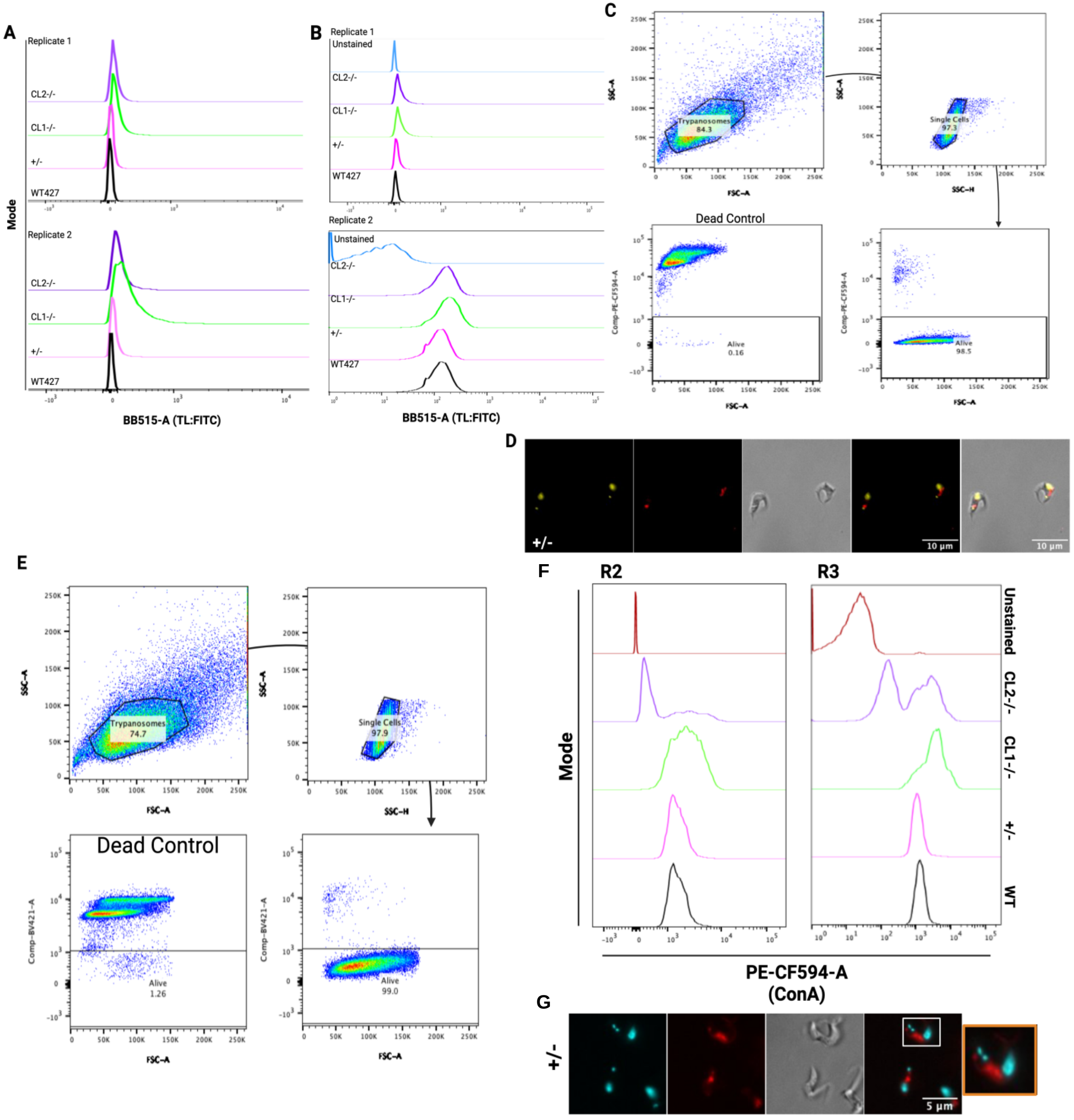
A
Replicate 1
CL2-/-
CL1-/-
+/-
WT427
Replicate 2
Mode
BB515-A (TL:FITC)
B
Unstained
C
Trypanosomes
84.3
Single Cells
97.3
FSC-A
SSC-A
SSC-H
Dead Control
Alive
0.16
98.5
Comp-PE-CF594-A
D
10 µm
E
74.7
97.9
Comp-BV421-A
1.26
99.0
F
R2
R3
WT
PE-CF594-A
(ConA)
G
5 µm

Figure S7

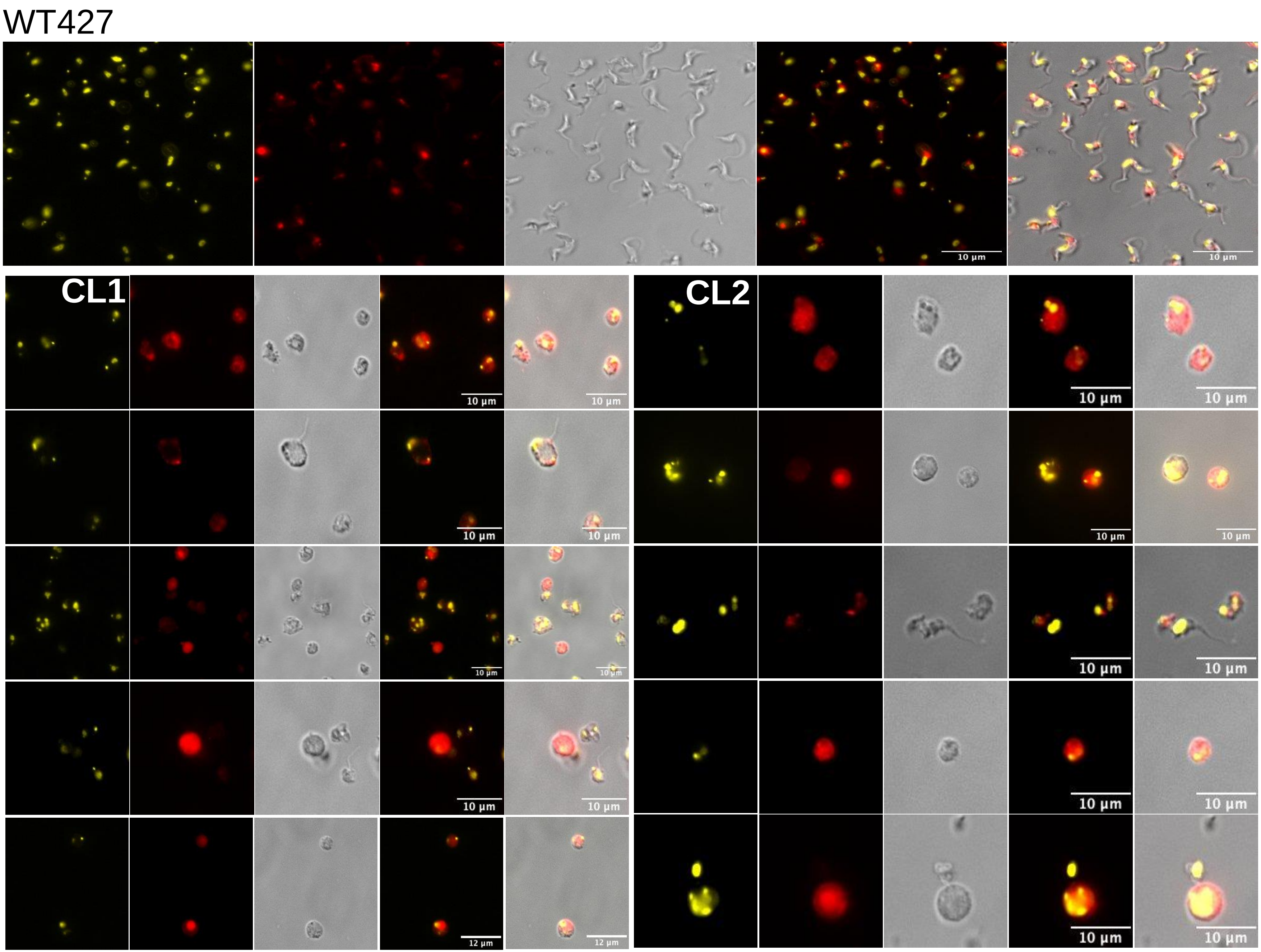

Supplementary Data 1

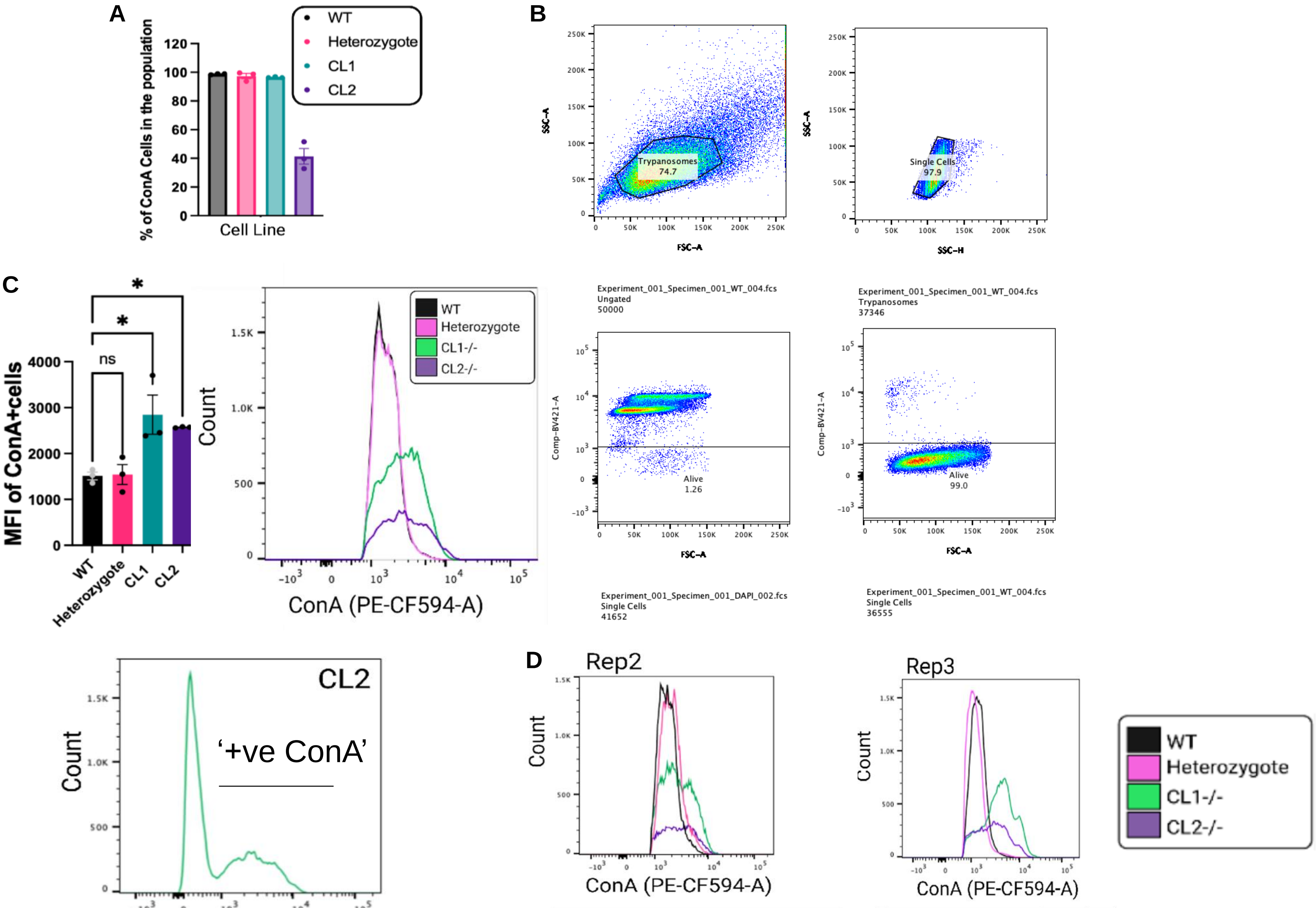

	Sample Name	Subset Name	Count
□	ConA_KOs_3-11-20_Specimen_001_Tube_005_005.fc	ConA+ve	44791
□	ConA_KOs_3-11-20_Specimen_001_Tube_006_006.fc	ConA+ve	46374
□	ConA_KOs_3-11-20_Specimen_001_Tube_007_007.fc	ConA+ve	41892
□	ConA_KOs_3-11-20_Specimen_001_Tube_008_008.fc	ConA+ve	14876

	Sample Name	Subset Name	Count
□	Experiment_001_Specimen_001_CL2_007.fcs	ConA+ve	21761
□	Experiment_001_Specimen_001_CL1_006.fcs	ConA+ve	39523
□	Experiment_001_Specimen_001_Hetero_005.fcs	ConA+ve	42000
□	Experiment_001_Specimen_001_WT_004.fcs	ConA+ve	41910

Supplementary Data 2
